# Supplementary material for: Role of chemical production and depositional losses on formaldehyde in the Community Regional Atmospheric Chemistry Multiphase Mechanism (CRACMM)
Source: Atmos Chem Phys. Author manuscript; Available in PMC 2025 Nov 21. (PMC12180760; doi:10.5194/acp-24-12903-2024)
Supplement: Supplement1 [file NIHMS2073166-supplement-Supplement1.pdf]

Supplement of Atmos. Chem. Phys., 24, 12903–12924, 2024
https://doi.org/10.5194/acp-24-12903-2024-supplement
© Author(s) 2024. CC BY 4.0 License.

Supplement of

Role of chemical production and depositional losses on formaldehyde in the Community Regional Atmospheric Chemistry Multiphase Mechanism (CRACMM)

T. Nash Skipper et al.

Correspondence to: Havala O.T. Pye (pye.havala@epa.gov)

The copyright of individual parts of the supplement might differ from the article licence.

Primary HCHO and total ROC emissions

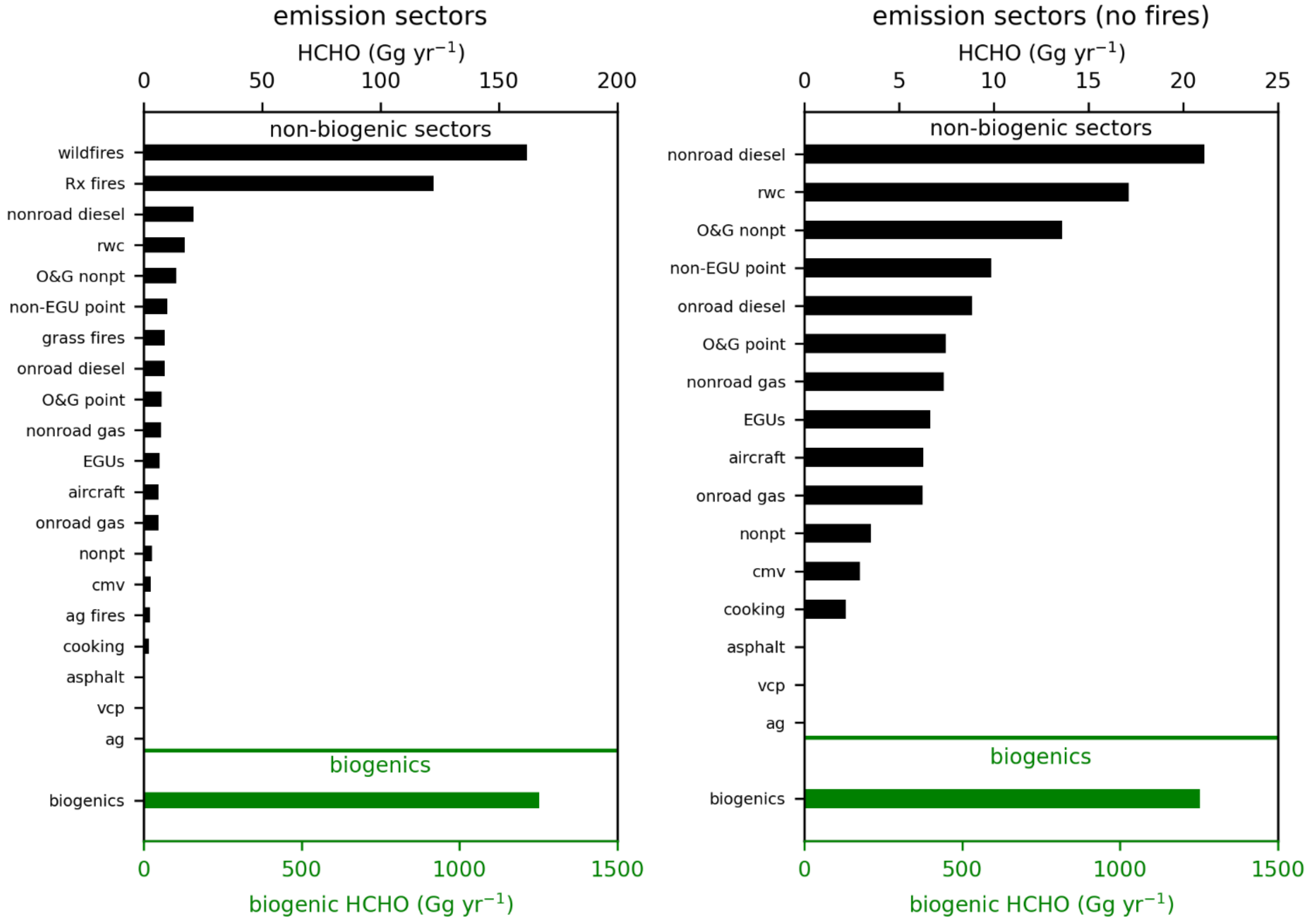

Figure S1. Primary HCHO by emission sector. Fires are included in the left panel and excluded on the right panel so that non-fire emission sector contributions can be seen in more detail.

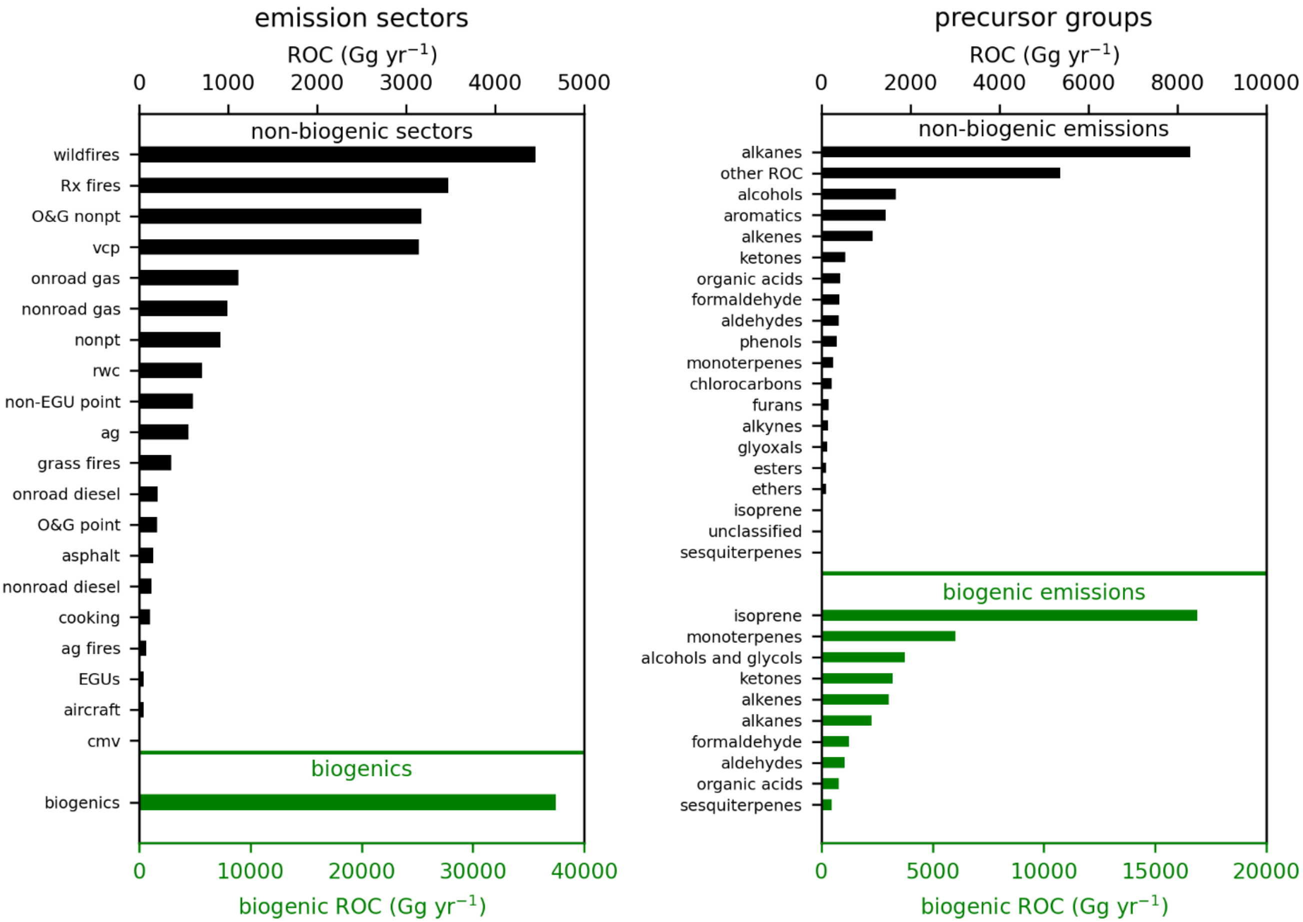

Figure S2. Total gas phase reactive organic carbon (ROC) emissions by emissions sector (left) and precursor group (right). Totals by emission sector (left) exclude primary HCHO which is shown separately in Fig. S1. Totals by precursor group (right) show primary HCHO separately from other aldehydes.

CRACMM2 mechanism updates

Table S1. Reactions updated in CRACMM2.

Reaction label	CRACMM1				CRACMM2				Note
	reactants		products	rate constant	reactants		products	rate constant	
**API system**									
R189	APIP1 + NO	-->	0.82*HO2 + 0.82*NO2 + 0.82*PINAL + 0.18*TRPN	4.00E-12	APIP1 + NO	-->	0.65*(0.23*TRPN + 0.77*HO2 + 0.77*NO2 + 0.3*PINAL + 0.27*ALD + 0.09*ACT + 0.09*LIMAL + 0.21*HCHO + 0.11*OPB) + 0.35*(0.08*ACT + 0.49*HCHO + 0.2*LIMAL + 0.24*KET + 0.25*TRPN + 0.31*ALD + 0.75*HO2 + 0.75*NO2)	2.70E-12*exp(360/T)	1, 3
R339	APIP1 + NO3	-->	HO2 + NO2 + ALD + KET	1.20E-12	APIP1 + NO3	-->	0.65*(NO2 + HO2 + 0.39*PINAL + 0.35*ALD + 0.12*ACT + 0.12*LIMAL + 0.27*HCHO + 0.14*OPB) + 0.35*(0.11*ACT + 0.65*HCHO + 0.27*LIMAL + 0.32*KET + 0.41*ALD + HO2 + NO2)	2.30E-12	1, 3
R229	APIP1 + HO2	-->	OPB	1.50E-11	APIP1 + HO2	-->	0.65*(0.06*ACT + 0.06*LIMAL + 0.08*HCHO + 0.65*OPB + 0.48*HO2 + 0.29*PINAL + 0.35*HO) + 0.35*(0.97*OPB + 0.03*HO + 0.03*KET + 0.03*HCHO + 0.03*HO2)	2.60E-13*exp(1300/T)	1, 3
R265	APIP1 + MO2	-->	HO2 + 0.68*HCHO + 0.60*PINAL + 0.07*KET + 0.32*MOH + 0.25*ROH	3.56E-14*exp(708/T)	APIP1 + MO2	-->	0.65*(0.83*HCHO + 0.14*LIMAL + 0.42*PINAL + 0.2*ALD + 0.13*OPB + 0.17*MOH + 0.11*KET + 0.06*ACT + 1.16*HO2) + 0.35*(1.4*HCHO + 0.37*LIMAL + 0.32*KET + 1.5*HO2 + 0.08*ACT + 0.31*ALD)	2.00E-12	1, 3
R301	APIP1 + ACO3	-->	0.63*HO2 + 0.70*MO2 + 0.60*PINAL + 0.30*ORA2 + 0.07*KET + 0.25*ROH	7.40E-13*exp(765/T)	APIP1 + ACO3	-->	0.65*(0.39*PINAL + 0.35*ALD + 0.14*OPB + 0.12*ACT + 0.12*LIMAL + 0.27*HCHO + HO2 + MO2) + 0.35*(0.32*KET + 0.27*LIMAL + 0.41*ALD + 0.11*ACT + 0.65*HCHO + HO2 + MO2)	2.00E-12*exp(500/T)	1, 3

Reaction label	CRACMM1				CRACMM2				Note
	reactants		products	rate constant	reactants		products	rate constant	
R131	API + O3	-->	0.90*HO + 0.90*APIP1 + 0.05*APIP2 + 0.05*PINAL + 0.05*H2O2 + 0.14*CO	5.00E-16*exp(-530/T)	API + O3	-->	0.65*(0.77*HO + 0.33*PINALP + 0.22*H2O2 + 0.39*PINAL + 0.01*ORA2 + 0.17*HO2 + 0.17*CO + 0.27*HCHO + 0.27*RCO3) + 0.35*(0.51*KET + 0.3*HO + 0.3*RCO3 + 0.32*H2O2 + 0.19*HC3 + 0.81*HCHO + 0.11*OP1 + 0.08*ORA1)	8.05E-16*exp(-640/T)	1, 3
TRP14	APINP1 + NO	-->	2.00*NO2 + PINAL	4.00E-12	APINP1 + NO	-->	0.65*(1.86*NO2 + 0.07*TRPN + 0.07*ONIT + 0.93*PINAL) + 0.35*(0.54*TRPN + 0.07*ONIT + 1.39*NO2 + 0.44*ALD + 0.02*KET + 0.02*HCHO + 0.47*HO2)	2.7E-12*exp(360/T)	1, 3
TRP53	---	-->	---	---	APINP1 + NO3	-->	0.65*(2*NO2 + PINAL) + 0.35*(1.5*NO2 + 0.5*TRPN + 0.48*ALD + 0.02*KET + 0.02*HCHO + 0.5*HO2)	2.30E-12	1, 3
TRP22	APINP1 + HO2	-->	TRPN	1.50E-11	APINP1 + HO2	-->	0.65*(0.3*TRPN + 0.7*PINAL + 0.7*NO2 + 0.7*HO) + 0.35*(0.47*HO + 0.76*TRPN + 0.22*ALD + 0.02*KET + 0.24*NO2 + 0.02*HCHO)	2.71E-13*exp(1300/T)	1, 3
TRP30	APINP1 + MO2	-->	0.37*HO2 + 0.86*NO2 + 0.68*HCHO + 0.86*PINAL + 0.32*MOH + 0.14*TRPN	3.56E-14*exp(708/T)	APINP1 + MO2	-->	0.65*(0.18*TRPN + 0.95*HCHO + 0.05*MOH + 0.82*HO2 + 0.82*NO2 + 0.82*PINAL) + 0.35*(0.64*TRPN + 0.02*KET + 0.34*ALD + 0.36*NO2 + 1.1*HO2 + 0.99*HCHO + 0.03*MOH)	2.00E-12	1, 3
TRP36	APINP1 + ACO3	-->	0.86*NO2 + 0.14*TRPN + 0.86*PINAL + 0.70*MO2 + 0.30*ORA2	7.40E-13*exp(765/T)	APINP1 + ACO3	-->	0.65*(NO2 + PINAL + MO2) + 0.35*(MO2 + 0.5*NO2 + 0.5*TRPN + 0.48*ALD + 0.02*KET + 0.02*HCHO + 0.5*HO2)	2.0E-12*exp(500/T)	1, 3
TRP19	PINALP + NO	-->	0.95*HO2 + 0.95*NO2 + 0.05*TRPN + 0.95*HCHO + 0.95*KET	2.70E-12*exp(360/T)	PINALP + NO	-->	0.36*TRPN + 0.64*HOM + 0.64*NO2	2.7E-12*exp(360/T)	1, 4, 5
TRP27	PINALP + HO2	-->	OPB	2.91E-13*exp(1300/T)	PINALP + HO2	-->	0.75*OPB + 0.25*HO + 0.25*HOM	2.71E-13*exp(1300/T)	1, 4, 5
TRP11	PINALP	-->	HOM	1	PINALP	-->	HOM	0.029	6
**LIM system**									
R190	LIMP1 + NO	-->	0.77*HO2 + 0.77*NO2 + 0.49*LIMAL + 0.28*HCHO + 0.28*UALD + 0.23*TRPN	4.00E-12	LIMP1 + NO	-->	0.23*TRPN + 0.77*NO2 + 0.77*LIMAL + 0.77*HO2 + 0.43*HCHO	2.70E-12*exp(360/T)	2, 3

Reaction label	CRACMM1				CRACMM2				Note
	reactants		products	rate constant	reactants		products	rate constant	
R340	LIMP1 + NO3	-->	HO2 + NO2 + 0.38500*OLI + 0.38500*HCHO + 0.61500*MACR	1.20E-12	LIMP1 + NO3	-->	NO2 + LIMAL + HO2 + 0.56*HCHO	2.30E-12	2, 3
R230	LIMP1 + HO2	-->	OPB	1.50E-11	LIMP1 + HO2	-->	0.9*OPB + 0.1*LIMAL + 0.1*HO + 0.1*HO2 + 0.06*HCHO	2.60E-13*exp(1300/T)	2, 3
R266	LIMP1 + MO2	-->	HO2 + HCHO + 0.42*LIMAL + 0.30*KET + 0.32*MOH + 0.27*ROH	3.560E-14*exp(708/T)	LIMP1 + MO2	-->	0.25*MOH + LIMAL + 1.03*HCHO + HO2	2.00E-12	2, 3
R302	LIMP1 + ACO3	-->	0.63*HO2 + 0.70*MO2 + 0.42*LIMAL + 0.30*KET + 0.30*ORA2 + 0.32*HCHO + 0.27*ROH	7.40E-13*exp(765/T)	LIMP1 + ACO3	-->	LIMAL + 0.56*HCHO + HO2 + MO2	2.00E-12*exp(500/T)	2, 3
R132	LIM + O3	-->	0.84*HO + 0.84*LIMP1 + 0.11*LIMP2 + 0.05*LIMAL + 0.05*H2O2 + 0.14*CO	2.95E-15*exp(-783/T)	LIM + O3	-->	0.66*HO + 0.66*LIMAL + 0.33*ACO3 + 0.33*HCHO + 0.33*RCO3 + 0.33*H2O2 + 0.01*ORA2	2.8E-15*exp(-770/T)	2, 3
TRP17	LIMNP1 + NO	-->	2.00*NO2 + LIMAL	4.00E-12	LIMNP1 + NO	-->	0.57*TRPN + 0.07*ONIT + 1.36*NO2 + 0.43*LIMAL + 0.5*HO2	2.7E-12*exp(360/T)	2, 3
TRP54	---	-->	---	---	LIMNP1 + NO3	-->	1.46*NO2 + 0.46*LIMAL + 0.54*TRPN + 0.54*HO2	2.30E-12	2, 3
TRP25	LIMNP1 + HO2	-->	TRPN	1.50E-11	LIMNP1 + HO2	-->	0.77*TRPN + 0.5*HO + 0.23*LIMAL + 0.23*NO2 + 0.27*HO2	2.71E-13*exp(1300/T)	2, 3
TRP33	LIMNP1 + MO2	-->	0.37*HO2 + 0.68*HCHO + 0.70*LIMAL + 0.70*NO2 + 0.32*MOH + 0.30*TRPN	3.56E-14*exp(708/T)	LIMNP1 + MO2	-->	0.69*TRPN + 0.91*HCHO + 0.09*MOH + 1.01*HO2 + 0.31*LIMAL + 0.31*NO2	2.00E-12	2, 3
TRP39	LIMNP1 + ACO3	-->	0.70*NO2 + 0.70*LIMAL + 0.30*TRPN + 0.70*MO2 + 0.30*ORA2	7.40E-13*exp(765/T)	LIMNP1 + ACO3	-->	MO2 + 0.46*NO2 + 0.46*LIMAL + 0.54*TRPN + 0.54*HO2	2.0E-12*exp(500/T)	2, 3
TRP05	LIMAL + HO	-->	0.70*LIMALP + 0.30*RCO3	1.00E-10	LIMAL + HO	-->	0.83*LIMALP + 0.17*RCO3	1.10E-10	2, 3
TRP20	LIMALP + NO	-->	0.94*HO2 + 0.94*NO2 + 0.06*TRPN + 0.94*HCHO + 0.94*KET	2.70E-12*exp(360/T)	LIMALP + NO	-->	0.64*TRPN + 0.36*NO2 + 0.36*HO2 + 0.36*HCHO + 0.36*PAA	2.7E-12*exp(360/T)	2, 4, 7
TRP28	LIMALP + HO2	-->	OPB	2.91E-13*exp(1300/T)	LIMALP + HO2	-->	0.9*OPB + 0.1*HO + 0.1*HO2 + 0.1*HCHO + 0.1*PAA	2.73E-13*exp(1300/T)	2, 4, 7
TRP12	LIMALP	-->	HOM	1	LIMALP	-->	HOM	0.024	6
TRP08	LIMAL + O3	-->	0.04*HO + 0.67*HC10P + 0.79*HCHO + 0.33*KET + 0.04*HO2 + 0.20*CO	8.30E-18	LIMAL + O3	-->	0.09*HO + ALD + 0.62*HCHO + 0.23*OP1 + 0.02*H2O2 + 0.15*ORA1	8.30E-18	2, 3
**Monoterpene nitrate system**									
TRP07	TRPN + HO	-->	HOM	4.80E-12	TRPN + HO	-->	0.33*HONIT + 0.67*NO2 + 0.27*PINAL + 0.38*KET + 0.21*HCHO + 0.02*ALD	4.80E-12	8
TRP09	TRPN + O3	-->	HOM	1.67E-16	TRPN + O3	-->	0.33*HONIT + 0.67*NO2 + 0.27*PINAL + 0.38*KET + 0.21*HCHO + 0.02*ALD	1.67E-16	8

Reaction label	CRACMM1				CRACMM2				Note
	reactants		products	rate constant	reactants		products	rate constant	
TRP10	TRPN + NO3	-->	HOM	3.15E-14*exp(-448/T)	TRPN + NO3	-->	0.33*HONIT + 0.67*NO2 + 0.27*PINAL + 0.38*KET + 0.21*HCHO + 0.02*ALD	3.15E-13*exp(-448/T)	8
TRP55	---	-->	---	---	TRPN + hv	-->	NO2 + 0.67*KET + 0.33*UALD	ONIT_RACM2	9
TRP56	---	-->	---	---	HONIT + hv	-->	HKET + NO2	ONIT_RACM2	9
TRP57	---	-->	---	---	HONIT + HO	-->	HKET + NO3	same as HNO3 + OH: xk0=2.40E-14*exp(460/T) xk2=2.70E-17*exp(2199/T) xk3=6.50E-34*exp(1335/T) k = xk0+xk3*M/(1.0+xk3*M/xk2)	10
TRP58	---	-->	---	---	ATRPNJ	-->	AHOMJ + HNO3	9.26E-05	11
TRP59	---	-->	---	---	AHONITJ	-->	AHOMJ + HNO3	9.26E-05	11
**STY system**									
ROCARO71	---	-->	---	---	STY + HO	-->	STYP	5.80E-11	12
ROCARO72	---	-->	---	---	STYP + HO2	-->	VROCP3OXY2	2.91E-13*exp(1300/T)	12, 13
ROCARO73	---	-->	---	---	STYP + NO	-->	NO2 + HO2 + HCHO + BALD	2.7E-12*exp(360/T)	12
ROCARO74	---	-->	---	---	STYP + NO3	-->	NO2 + HO2 + HCHO + BALD	2.30E-12	12
ROCARO75	---	-->	---	---	STYP + MO2	-->	HO2 + HCHO + BALD + 0.68*HCHO + 0.37*HO2 + 0.32*MOH	2.50E-13	12
ROCARO76	---	-->	---	---	STYP + ACO3	-->	HO2 + HCHO + BALD + 0.7*MO2 + 0.3*ORA2	2.50E-13	12
**ISO system**									
R130/RAM01	ISO + O3	-->	0.25*HO + 0.25*HO2 + 0.08*MO2 + 0.10*ACO3 + 0.10*MACP + 0.09*H2O2 + 0.14*CO + 0.58*HCHO + 0.46100*MACR + 0.18900*MVK + 0.28*ORA1 + 0.15300*OLT	7.86E-15*exp(-1913/T)	ISO + O3	-->	0.25*HO + 0.25*HO2 + 0.40*MO2 + 0.01800*ACO3 + 0.10*MACP + 0.09*H2O2 + 0.22*CO + HCHO + 0.30*MACR + 0.14*MVK + 0.28*ORA1 + 0.15300*OLT	1.58E-14*exp(-2000/T)	14
R145/RAM02	ISO + NO3	-->	ISON	3.03E-12*exp(-446/T)	ISO + NO3	-->	0.40*NO2 + 0.04500*ISON + 0.35*HCHO + 0.55500*INO2 + 0.26*MVK + 0.02800*MACR	2.95E-12*exp(-450/T)	14
R086/RAM03	ISO + HO	-->	ISOP	2.70E-11*exp(390/T)	ISO + HO	-->	ISOP + 0.25*HCHO + 0.03*MACR + 0.05*MGLY	2.69E-11*exp(390/T)	14
R228/RAM04	ISOP + HO2	-->	ISHP	2.05E-13*exp(1300/T)	ISOP + HO2	-->	ISHP + 0.07*HO2 + 0.50*HO	4.50E-13*exp(1300/T)	14
R188/RAM05	ISOP + NO	-->	0.88*HO2 + 0.88*NO2 + 0.20*HCHO + 0.28*MACR + 0.44*MVK + 0.12*ISON + 0.02100*GLY + 0.02900*HKET + 0.02700*ALD	2.43E-12*exp(360/T)	ISOP + NO	-->	0.13*ISON + 0.40*HCHO + 0.88*HO2 + 0.87*NO2 + 0.18*MACR + 0.51*MVK	6.00E-12*exp(350/T)	14
R115/RAM06	ISHP + HO	-->	HO + MACR + 0.90400*IEPOX	1.00E-10	ISHP + HO	-->	ISOP	4.60E-12*exp(200/T)	14

Reaction label	CRACMM1				CRACMM2				Note
	reactants		products	rate constant	reactants		products	rate constant	
RAM07	---	-->	---	---	ISHP + HO	-->	0.04*MGLY + 0.02*GLY + 0.13*MVK + 0.44*IEPOX + 0.11*ACO3 + 0.03*MACR + 2.00*HO + 0.34*HO2 + 0.14*IPX + CO	2.97E-11*exp(390/T)	14
RAM08	---	-->	---	---	INO2 + HO2	-->	0.45*HO + 0.95*INALD + 0.02*IPX	3.14E-11*exp(580/T)	14
RAM09	---	-->	---	---	INO2 + NO	-->	0.15*MVK + 0.65*INALD + 0.05*ISON + 0.20*HCHO + 1.30*NO2	9.42E-12*exp(580/T)	14
R125/RAM10	ISON + HO	-->	INALD + 0.07*HKET + 0.07*HCHO	1.30E-11	ISON + HO	-->	HO + 0.35*INALD + 0.17*IEPOX + 0.65*NO2	2.40E-11*exp(390/T)	14
R124/RAM11	NALD + HO	-->	NO2 + XO2 + HKET	5.60E-12*exp(270/T)	INALD + HO	-->	CO + NO2 + 0.30*HO2 + HCHO	1.50E-11	14
RAM12	---	-->	---	---	ISON	-->	HNO3 + ROH	4.00E-05	14
RAM13	---	-->	---	---	IPX + HO	-->	0.57*MACR + 0.43*MVK	3.00E-12	14
**Hetereogeneous reactions**									
HET_IPX	---	-->	---	---	IPX	-->	AISO4J	$2*k_{het\ IEPOX}$	15
HET_INALD	---	-->	---	---	INALD	-->	AISO5J + HNO3	$0.5*k_{het\ IEPOX}$	15
HET_HO2	---	-->	---	---	HO2	-->	H2O	$k_{het\ HO2}$ (γ = 0.2)	16
HET_NO3	---	-->	---	---	NO3	-->	HNO3	$k_{het\ NO3}$ (γ = 1E-3)	17
**Methane**									
R364	---	-->	---	---	ECH4 + HO	-->	MO2	2.45E-12*exp(-1775/T)	18

Notes for Table S1.

1. Products in the API system have been updated to include products from α-pinene and β-pinene, assuming a 65/35 split of α-/β-pinene (based on biogenic emission totals over the contiguous US). Updated reactions are based on α-pinene and β-pinene chemistry in the MOZART-TS2 mechanism developed by Schwantes et al. (2020).

2. Reactions and products in the LIM system have been updated based on limonene chemistry in the MOZART-TS2 mechanism developed by Schwantes et al. (2020).

3. Species from MOZART-TS2 have been mapped to existing CRACMM species. Mapping from MOZART-TS2 to CRACMM2 includes the following:
    a. TS2 monoterpene nitrate species were mapped to CRACMM species TRPN.
        i. Except TS2 species TERPFDN (monoterpene derived di-nitrate) is mapped to CRACMM species TRPN+ONIT for conservation of nitrogen.
    b. TS2 species TERPA is mapped to CRACMM species PINAL.
    c. TS2 species TERPA3 is mapped to CRACMM species ALD.
    d. TS2 species TERPF1 is mapped to CRACMM species LIMAL.
    e. TS2 species TERP1OOH and TERPOOH are mapped to CRACMM species OPB.
    f. TS2 species TERPK is mapped to CRACMM species KET.
    g. TS2 species TERPA2O2 is mapped to CRACMM species PINALP.
    h. TS2 species TERPACID is mapped to CRACMM species ORA2.
    i. TS2 species TERPA2 is mapped to CRACMM species PINAL.
    j. TS2 species TERPA2CO3 is mapped to CRACMM species RCO3.
    k. TS2 species BIGALK is mapped to CRACMM species HC3.
    l. TS2 species HMHP is mapped to CRACMM species OP1.

4. The following characteristics of monoterpene aldehyde derived peroxy radical (PINALP and LIMALP) reactions are based on Wennberg et al. (2018):
    a. Nitrate (CRACMM species TRPN) branching ratio from reaction with NO
    b. Rate of reaction with HO2
    c. Yield of peroxide (CRACMM species OPB) from reaction with HO2

5. Products of PINALP reaction with NO and HO2 that do not go to TRPN or OPB, respectively, are expected to form a ring-opening peroxy radical that undergoes autoxidation which is mapped to CRACMM species HOM.

6. Unimolecular autoxidation rates of PINALP and LIMALP are calculated based on Vereecken and Nozière (2020) and assuming that the first competitive step rather than the first step best reflects the rate of HOM formation in a reduced mechanism. Details:
    a. For PINALP rate:
        i. From Table 1 k(298 K) = 6.2E-4 s^{-1} (1,5-H migration; substitution pattern –CH< >C(OO•)-)
        ii. From Table 5 k(298 K) correction factor = 47 (exo-β-oxo 1,5)
        iii. Overall k = 6.2E-4 × 47 = 0.029 s^{-1}
    b. For LIMALP rate both 1,5-H shift and 1,6-H shift are possible. We consider both options and add the rate constant from the two options to calculate the overall rate constant:
        i. From Table1:
            1. k_1(298 K) = 4.49E-4 s^{-1} (1,5-H migration; substitution pattern –CH_2– >C(OO•)-)
            2. k_2(298 K) = 3.12E-4 s^{-1} (1,6-H migration; substitution pattern –CH_2– >C(OO•)-)
        ii. From Table 5:
            1. k_1(298 K) correction factor = 47 (exo-β-oxo 1,5)
            2. k_2(298 K) correction factor = 10 (exo-β-oxo 1,6)
        iii. Overall k = 4.49E-4 × 47 + 3.12E-4 × 10 = 0.024 s^{-1}

7. Products of LIMALP reaction with NO and HO2 that do not go to TRPN or OPB, respectively, are expected to form a peroxy acid (mapped to CRACMM species PAA) and formaldehyde (CRACMM species HCHO).

8. Oxidation of TRPN assumes 1/3 is unsaturated and retains the nitrate group upon oxidation to from HONIT while the remaining 2/3 is saturated and releases the nitrate group to form NO2 and other products. The 1/3 to 2/3 split is based on Fisher et al. (2016). The coefficients of other products are based on products from α-pinene and β-pinene derived nitrates in MCM with a 65/35 weighting as described in Note 1 above. From MCM products, we find a 62% yield of PINAL and a 38% yield of a ketone (KET) for α-pinene and a 92% yield of a ketone (KET) + HCHO and an 8% yield of an aldehyde (ALD) for β-pinene.

9. Photolysis rates of monoterpene nitrates are set to the same rate as the other organic nitrate species in CRACMM (ONIT). Photolysis products are NO2 and fragmentation products.

10. Oxidation of HONIT is based on Fisher et al. (2016) and Browne et al. (2014).

11. ATRPN and AHONIT are expected to undergo hydrolysis with a 3-h lifetime based on Pye et al. (2015).

12. Styrene chemistry is adopted from MCM for CRACMM2.

13. MCM predicts a peroxide formed from the reaction of the styrene peroxy radical with HO2. In CRACMM2, this peroxide is mapped to an oxygenated IVOC with C*=10^3 µg/m^3 and O:C=0.2 (CRACMM species VROCP3OXY2).

14. From AMOREv1.2 isoprene condensation of a detailed isoprene mechanism by Wennberg et al. (2018). For more details on the initial development of the AMORE technique and condensed isoprene mechanism (i.e., AMOREv1.0), Wiser et al. (2023). For more details specific to AMOREv1.2, see Section 2.1 of the main text.

15. Heterogeneous uptake of isoprene derived nitrates (INALD) and isoprene derived tetrafunctional compounds (IPX) are set to be proportional to IEPOX uptake.

16. HO2 radical uptake is based on Ivatt et al. (2022).

17. NO3 radical uptake is based on Jacob (2000) and Zhu et al. (2024).

18. Emitted methane (ECH4) is tracked separately to account for effects of methane emissions on top of the global average background methane (set to 1850 ppb in CMAQ by default).

AMORE v1.2 updates overview

The AMORE v1.0 Isoprene Mechanism was developed from the Caltech Isoprene Mechanism using a graph theory-based algorithm for the reduction of large chemical mechanisms (Wiser et al., 2023). This mechanism was added as an option in CRACMM1. Subsequent updates were made from new GEOS-Chem data (Yang et al., 2023), CMAQ data and box model results (this work). The updated AMORE v1.2 isoprene mechanism is included with this mechanism update, and includes improved NOx chemistry, improved VOC yields, including formaldehyde, and additional SOA pathways from isoprene derived tetrafunctional compounds.

GEOS-Chem simulation results showed that the AMORE v1.0 mechanism had elevated NOx production leading to increased ozone bias. This was confirmed with CMAQ simulations and replicated in box model simulations, and the NOx chemistry was adjusted by reducing the yield of NO2 to better represent the full reference mechanism, leading to improved NOx chemistry and reduced ozone production, which resulted in stronger model agreement with atmospheric data. For further information, see Yang et al. (2023).

CMAQ simulations showed that the AMORE v1.0 formaldehyde yield was notably higher than base CRACMM and higher than TROPOMI measurements, despite the AMORE v1.0 mechanism having strong agreement with box model runs. We found that the cause of this discrepancy was in the tail end production of formaldehyde, which occurs at timescales on the order of multiple days. This long tail formaldehyde production was captured in select box model runs but not in measured data, where transport and condensation would likely limit tail end formaldehyde production. Running the box model with diurnal cycle data reduced the discrepancy between the box model and the measured data. The primary source of formaldehyde in the AMORE mechanism is from the reaction of isoprene with OH. The molar yield of formaldehyde from this reaction was adjusted to determine which value best represented the full mechanism and the intended use in transport models. As shown in Fig. S3, in a diurnal cycle run, the 0.25 molar yield mechanism has much stronger agreement with the reference mechanism after one day. From two to three days, the mechanisms begin to diverge, with the reference mechanism having a higher formaldehyde yield. This figure shows the overall net production of formaldehyde, as dilution and formaldehyde decomposition reactions were turned off.

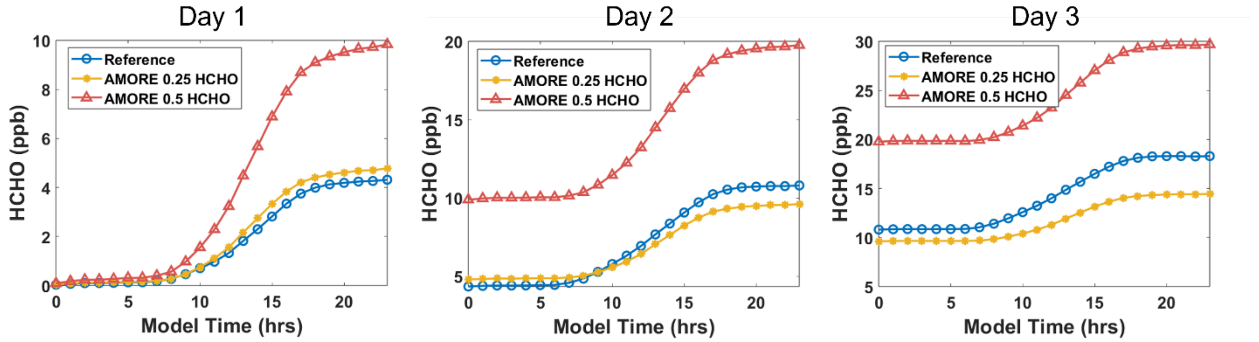

Figure S3. Concentration of formaldehyde for the 0.25 molar yield AMORE mechanism vs. the 0.5 molar yield AMORE mechanism in comparison to the Caltech Full Reference Isoprene mechanism. These plots show three consecutive days using the F0AM diurnal cycle run with hourly concentration data for isoprene and reactive atmospheric gases along with solar intensity values from 6/30/2013 in Centerville, Alabama. Data is from the SOAS field campaign. Ozone and NOx concentrations are set to zero to focus on the primary formaldehyde pathway from isoprene reacting with OH. Dilution is set to zero and formaldehyde decomposition reactions are removed to demonstrate accumulated yield of formaldehyde over the entire run.

We conducted a run with several different formaldehyde molar yields in CMAQ, ranging from 0.25 to 0.5. The results are shown in Fig. S4, with comparisons to the CRACMM1 baseline mechanism and TROPOMI data. All AMORE mechanisms showed increased formaldehyde production from the CRACMM1 baseline mechanism. The CRACMM1 baseline mechanism had negative formaldehyde bias compared to the TROPOMI data. The overall bias was reduced for each AMORE mechanism except for the 0.5 molar yield mechanism, which had significant positive formaldehyde bias.

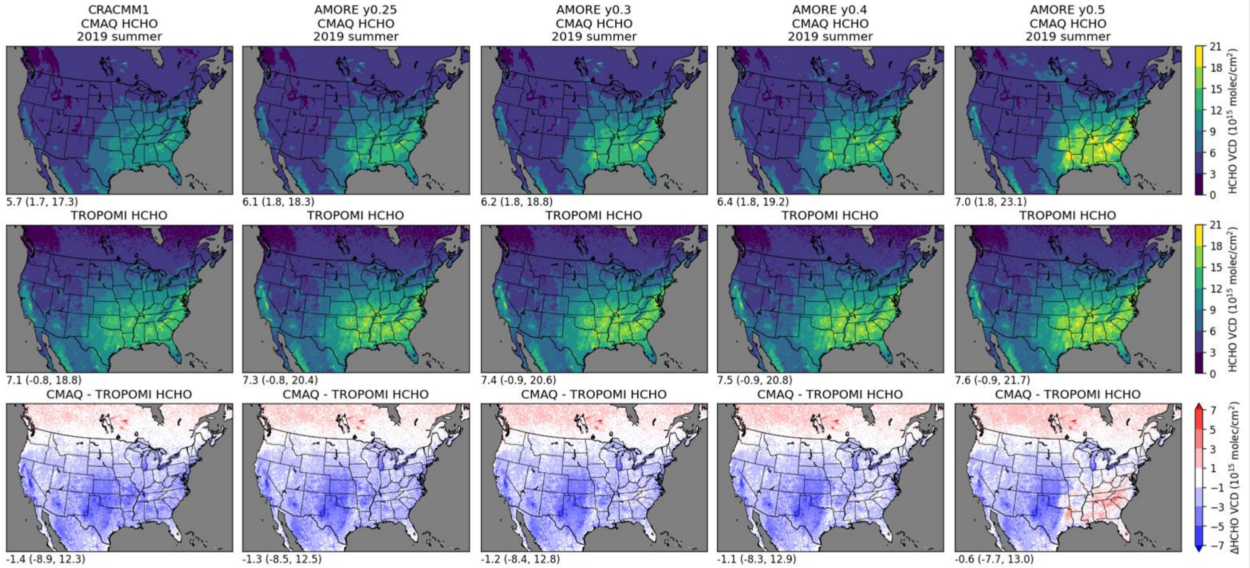

Figure S4. CMAQ simulations of 4 different formaldehyde yields for the AMORE mechanism, in comparison to the CRACMM1 baseline mechanism and TROPOMI data. The simulation and data are taken from the summer of 2019. The first row shows the simulated concentration of formaldehyde for each mechanism. The second row shows the measured TROPOMI values, and the final row shows the bias between the simulation and the TROPOMI values.

Ultimately, the TROPOMI data is a useful comparison point but cannot be used to calibrate the formaldehyde yield, as there is no way to isolate isoprene derived formaldehyde from this data. According to the box model results, any discrepancies between the AMORE 0.25 yield mechanism and the reference mechanism would only show up after 2 days, and only reach significant levels after 3 days. Most of this tail end formaldehyde production is the result of aged, highly oxidized isoprene derived species, which would suggest that other processes such as deposition, condensation, and transport might dominate at this timescale, leading to suppressed production of formaldehyde in the gas phase. Thus, the 0.25 formaldehyde molar yield was chosen for the AMORE v1.2 mechanism used in this work. We made similar adjustments to other VOC species such as methylglyoxal and glyoxal.

Isoprene derived tetrafunctional compounds, including multifunctional epoxydiols which are similar but distinct from isoprene epoxy-diol, are potentially significant sources of SOA from isoprene (Bates et al). They estimate that IEPOX, organonitrates, and isoprene tetrafunctional compounds all contribute approximately 30% each of isoprene derived SOA. Of these, isoprene tetrafunctional compounds have the least studied chemistry, resulting in some uncertainty in their SOA yields. A lumped species was added to the AMORE v1.2 isoprene mechanism to represent this group, and an SOA formation pathway was added with the same mechanism as IEPOX, but with a lower SOA yield due to uncertainties in the behavior of these species. The molar yield of this new species was determined by comparison to the set of tetrafunctional compounds represented in the Caltech full isoprene mechanism. The concentration plot comparison is shown in Fig. S7.

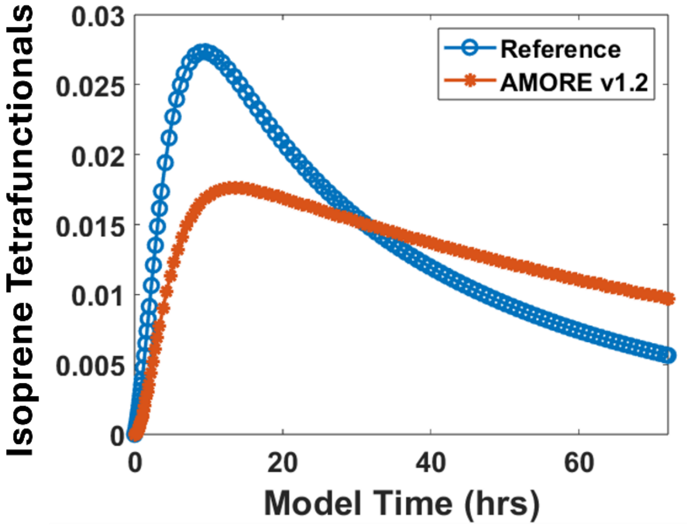

Figure S5. This figure shows the concentration of isoprene tetrafunctional compounds for the AMORE v1.2 mechanism and the Caltech reference full isoprene mechanism over a 72-hour F0AM box model run with moderate light conditions, 1 ppb of isoprene, 4e-5 ppb OH, 10 ppb O_3, 0.005 ppb HO2, and 0.05 ppb NO.

Pinene biogenic emissions

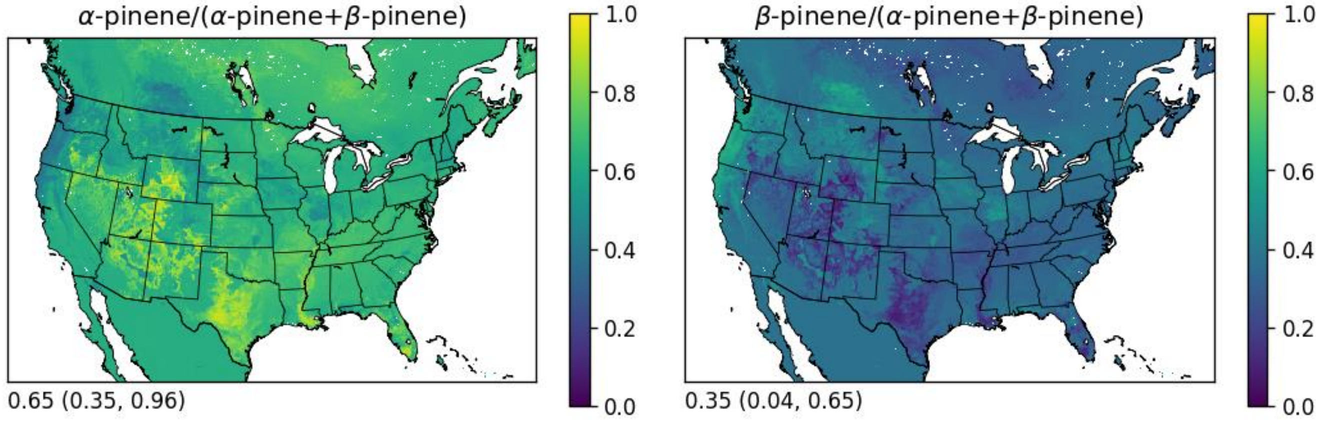

Figure S6. Ratio of α-pinene and β-pinene to total pinenes based on the annual sum of biogenic emissions from BEIS. The values for α-pinene and β-pinene include additional monoterpene emissions from BEIS that would map to these species. Monoterpenes mapped to β-pinene are those that have one double bond which is a terminal double bond and include β -pinene, camphene, and sabinene. Monoterpenes mapped to α-pinene are all other monoterpenes with one double bond that is not terminal and include α-pinene, δ-3-carene, and α-thujene. The annotations below each panel show the mean (min, max) over land grid cells.

Additional information on F0AM simulations

Table S2. Description of each emission sector used in the F0AM box modeling simulations. Most descriptions are based on information provided with the 2019 Emissions Modeling Platform (U.S. EPA, 2022).

Sector	Description
biogenics	Emissions from BEIS; typically simulated in-line in CMAQ
wildfires	Wildfire emissions
Rx fires	Prescribed (Rx) burning emissions
grass fires	Grassland burning emissions
ag fires	Agricultural burning emissions
rwc	Residential wood combustion emissions
O&G point	Point source oil and gas emissions
O&G nonpt	Area source oil and gas emissions
vcp	Volatile chemical product emissions
onroad gas	Highway mobile source emissions from gasoline vehicles
nonroad gas	Off highway mobile source emissions from gasoline vehicles
onroad diesel	Highway mobile source emissions from diesel vehicles
nonroad diesel	Off highway mobile source emissions from diesel vehicles
nonpt	Area source emissions not included in other sectors
non-EGU point	Point source emissions from industrial activities (also known as ptnonipm)
ag	Agricultural emissions
asphalt	Asphalt paving emissions
cooking	Cooking emissions
EGUs	Electric generating unit emissions
aircraft	Aircraft emissions
cmv	Class 1, 2, and 3 commercial marine vessel emissions

Table S3. Precursor groups used in F0AM simulations. The MCM column indicates the MCM species included in each group. The CRACMM1 and CRACMM2 columns indicate the species from those mechanisms that best corresponds with each MCM species. Species mappings that differ in CRACMM1 and CRACMM2 are colored blue.

group	MCM	CRACMM1	CRACMM2
alcohols	CH3OH	MOH	MOH
	C2H5OH	EOH	EOH
	NPROPOL	ROH	ROH
	IPROPOL	ROH	ROH
	NBUTOL	ROH	ROH
	BUT2OL	ROH	ROH
	IBUTOL	ROH	ROH
	TBUTOL	ROH	ROH
	PECOH	ROH	ROH
	IPEAOH	ROH	ROH
	ME3BUOL	ROH	ROH
	IPECOH	ROH	ROH
	IPEBOH	ROH	ROH

group	MCM	CRACMM1	CRACMM2
	CYHEXOL	ROH	ROH
	MIBKAOH	HKET	HKET
	ETHGLY	ETEG	ETEG
	PROPGLY	PROG	PROG
	MBO	OLT	OLT
formaldehyde	HCHO	HCHO	HCHO
aldehydes	CH3CHO	ACD	ACD
	C2H5CHO	ALD	ALD
	C3H7CHO	ALD	ALD
	IPRCHO	ALD	ALD
	C4H9CHO	ALD	ALD
	ACR	ACRO	ACRO
	MACR	MACR	MACR
	C4ALDB	MACR	MACR
alkanes	CH4	n/a	ECH4
	C2H6	ETH	ETH
	C3H8	HC3	HC3
	NC4H10	HC3	HC3
	IC4H10	HC3	HC3
	NC5H12	HC5	HC5
	IC5H12	HC5	HC5
	NEOP	HC3	HC3
	NC6H14	HC5	HC5
	M2PE	HC5	HC5
	M3PE	HC5	HC5
	M22C4	HC3	HC3
	M23C4	HC5	HC5
	NC7H16	HC10	HC10
	M2HEX	HC5	HC5
	M3HEX	HC5	HC5
	NC8H18	HC10	HC10
	NC9H20	HC10	HC10
	NC10H22	HC10	HC10
	NC11H24	HC10	HC10
	NC12H26	ROCP6ALK	ROCP6ALK
	CHEX	HC10	HC10
alkenes	C2H4	ETE	ETE
	C3H6	OLT	OLT
	BUT1ENE	OLT	OLT
	CBUT2ENE	OLI	OLI
	TBUT2ENE	OLI	OLI
	MEPROPENE	OLT	OLT

group	MCM	CRACMM1	CRACMM2
	PENT1ENE	OLT	OLT
	CPENT2ENE	OLI	OLI
	TPENT2ENE	OLI	OLI
	ME2BUT1ENE	OLT	OLT
	ME3BUT1ENE	OLT	OLT
	ME2BUT2ENE	OLI	OLI
	HEX1ENE	OLT	OLT
	CHEX2ENE	OLI	OLI
	THEX2ENE	OLI	OLI
	DM23BU2ENE	OLI	OLI
	C4H6	BDE13	BDE13
alkynes	C2H2	ACE	ACE
aromatics	BENZENE	BEN	BEN
	TOLUENE	TOL	TOL
	OXYL	XYE	XYL
	MXYL	XYM	XYL
	PXYL	XYE	XYL
	EBENZ	XYE	EBZ
	PBENZ	XYE	XYL
	IPBENZ	XYE	XYL
	TM123B	XYM	XYL
	TM124B	XYM	XYL
	TM135B	XYM	XYL
	OETHTOL	XYE	XYL
	METHTOL	XYM	XYL
	PETHTOL	XYE	XYL
	DIME35EB	XYE	XYL
	DIET35TOL	XYM	XYL
	STYRENE	XYM	STY
	BENZAL	BALD	BALD
	MCATECHOL	MCT	MCT
	n/a (use MXYL)	NAPH	NAPH
chlorocarbons	CH3CL	SLOWROC	SLOWROC
	CH2CL2	SLOWROC	SLOWROC
	CHCL3	SLOWROC	SLOWROC
	CH3CCL3	SLOWROC	SLOWROC
	TCE	SLOWROC	SLOWROC
	TRICLETH	OLI	OLI
	CDICLETH	OLI	OLI
	TDICLETH	OLI	OLI
	CH2CLCH2CL	SLOWROC	SLOWROC

group	MCM	CRACMM1	CRACMM2
	CCL2CH2	OLT	OLT
	CL12PROP	HC3	HC3
	CHCL2CH3	SLOWROC	SLOWROC
	CH3CH2CL	HC3	HC3
	CHCL2CHCL2	SLOWROC	SLOWROC
	CH2CLCHCL2	SLOWROC	SLOWROC
	VINCL	OLT	OLT
isoprene	C5H8	ISO	ISO
esters	CH3OCHO	SLOWROC	SLOWROC
	METHACET	SLOWROC	SLOWROC
	ETHACET	HC3	HC3
	NPROACET	HC3	HC3
	IPROACET	HC3	HC3
	NBUTACET	HC5	HC5
	SBUTACET	HC5	HC5
	TBUACET	HC3	HC3
ethers	CH3OCH3	HC3	HC3
	DIETETHER	HC10	HC10
	MTBE	HC3	HC3
	DIIPRETHER	HC10	HC10
	ETBE	HC10	HC10
	MO2EOL	ROH	ROH
	EOX2EOL	ROH	ROH
	PR2OHMOX	ROH	ROH
	BUOX2ETOH	ROH	ROH
	BOX2PROL	ROH	ROH
ketones	CH3COCH3	ACT	ACT
	MEK	MEK	MEK
	MPRK	KET	KET
	DIEK	KET	KET
	MIPK	KET	KET
	HEX2ONE	KET	KET
	HEX3ONE	KET	KET
	MIBK	KET	KET
	MTBK	KET	KET
	CYHEXONE	KET	KET
	MVK	MVK	MVK
monoterpenes	APINENE	API	API
	BPINENE	API	API
	LIMONENE	LIM	LIM
sesquiterpenes	BCARY	SESQ	SESQ
organic acids	HCOOH	ORA1	ORA1

group	MCM	CRACMM1	CRACMM2
	CH3CO2H	ORA2	ORA2
	PROPACID	ORA2	ORA2
unclassified	DMM	HC5	HC5
	DMC	SLOWROC	SLOWROC
	DMS	HC5	HC5
	ETHOX	SLOWROC	SLOWROC
phenols	CRESOL	CSL	CSL
	PHENOL	PHEN	PHEN
furans	PXYFUONE	FURAN	FURAN
glyoxals	GLYOX	GLY	GLY
	MGLYOX	MGLY	MGLY

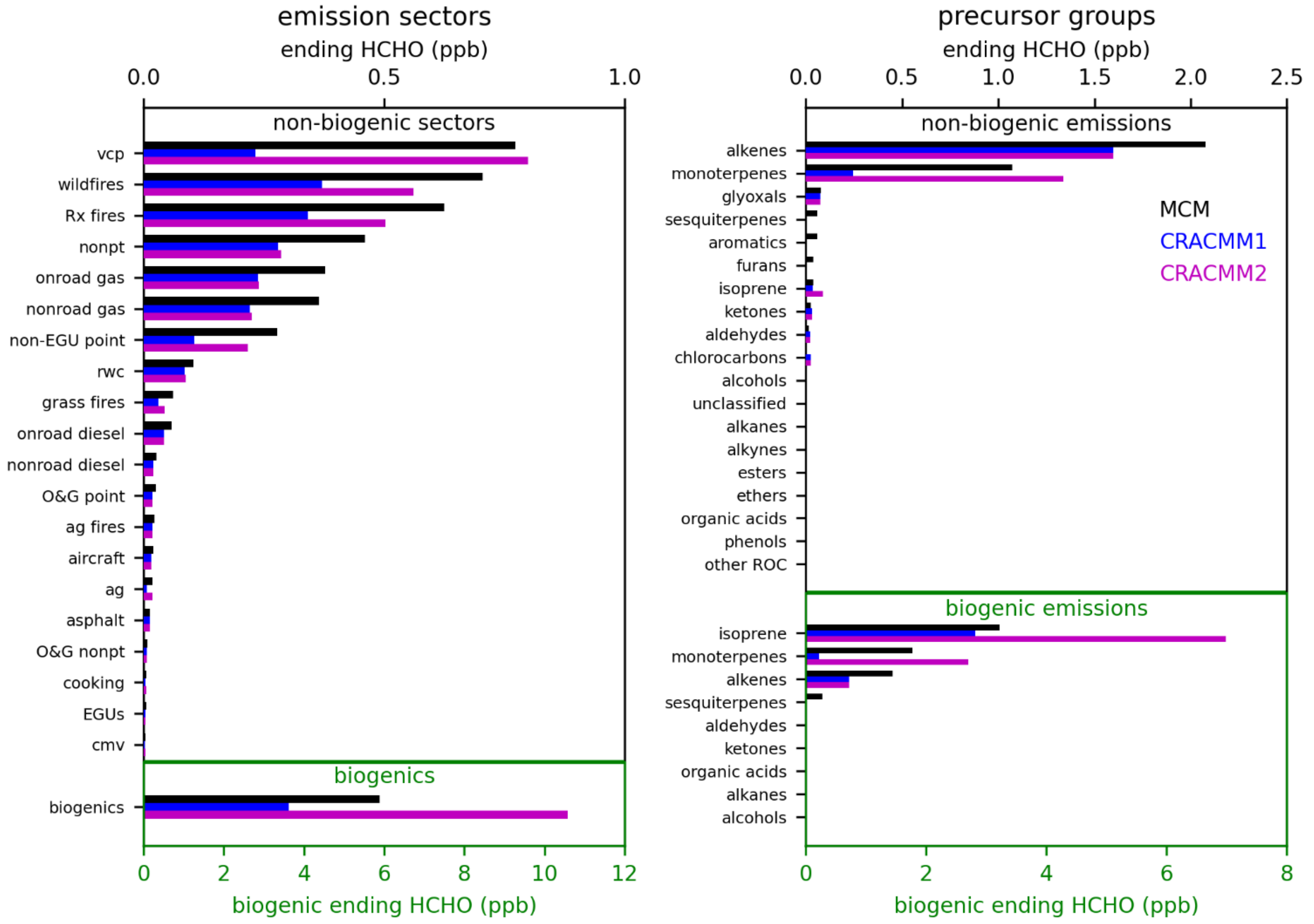

Figure S7. Like Figure 1, except that the F0AM simulation holds OH constant at zero and holds ozone constant at 30 ppb. Several categories of precursors have no ozonolysis channel so do not produce any HCHO in these simulations.

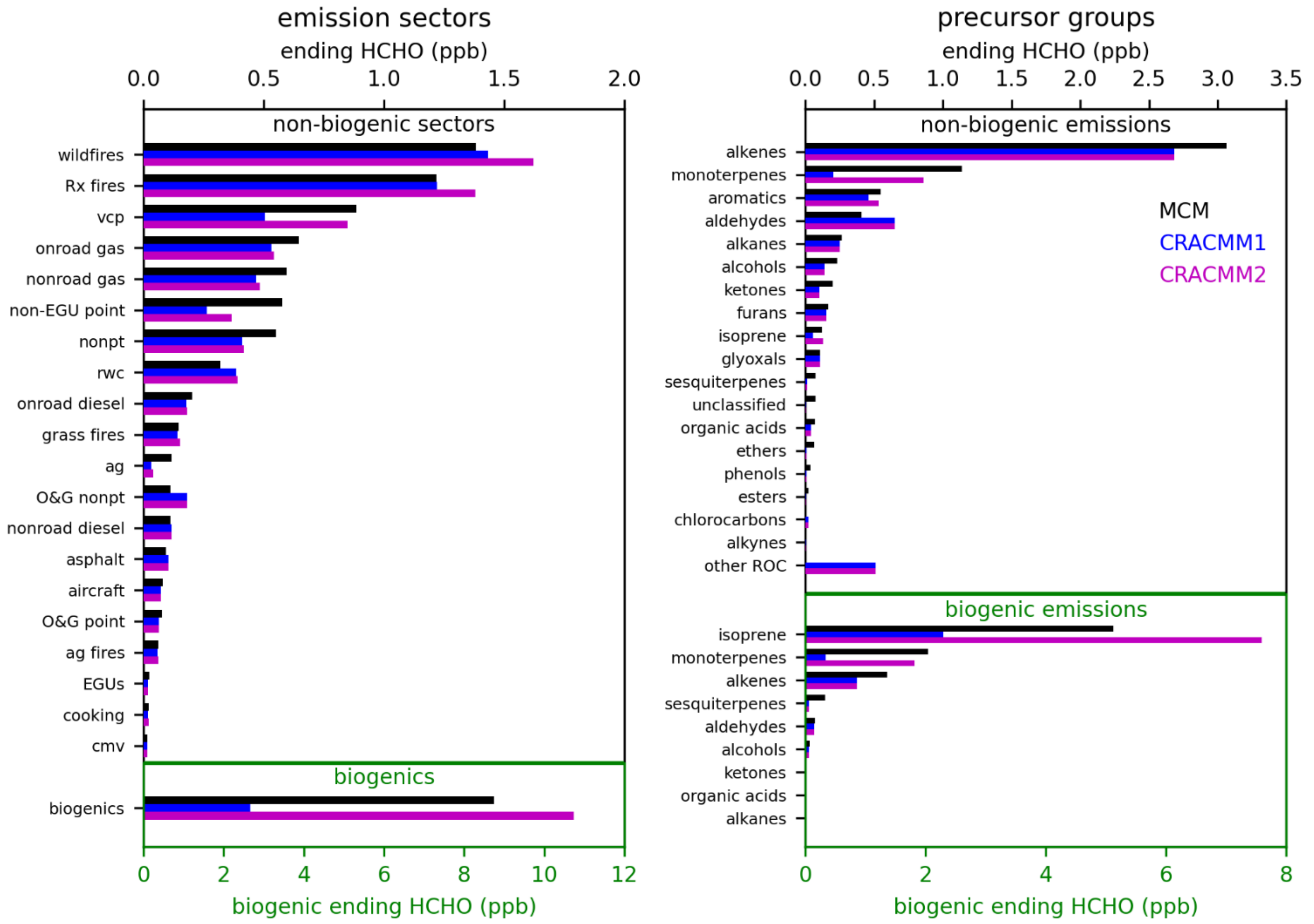

Figure S8. Like Figure 1, except that the F0AM simulation holds OH constant at 10^6 molecules cm^{-3} and holds ozone constant at 30 ppb.

Additional CMAQ surface concentration plots

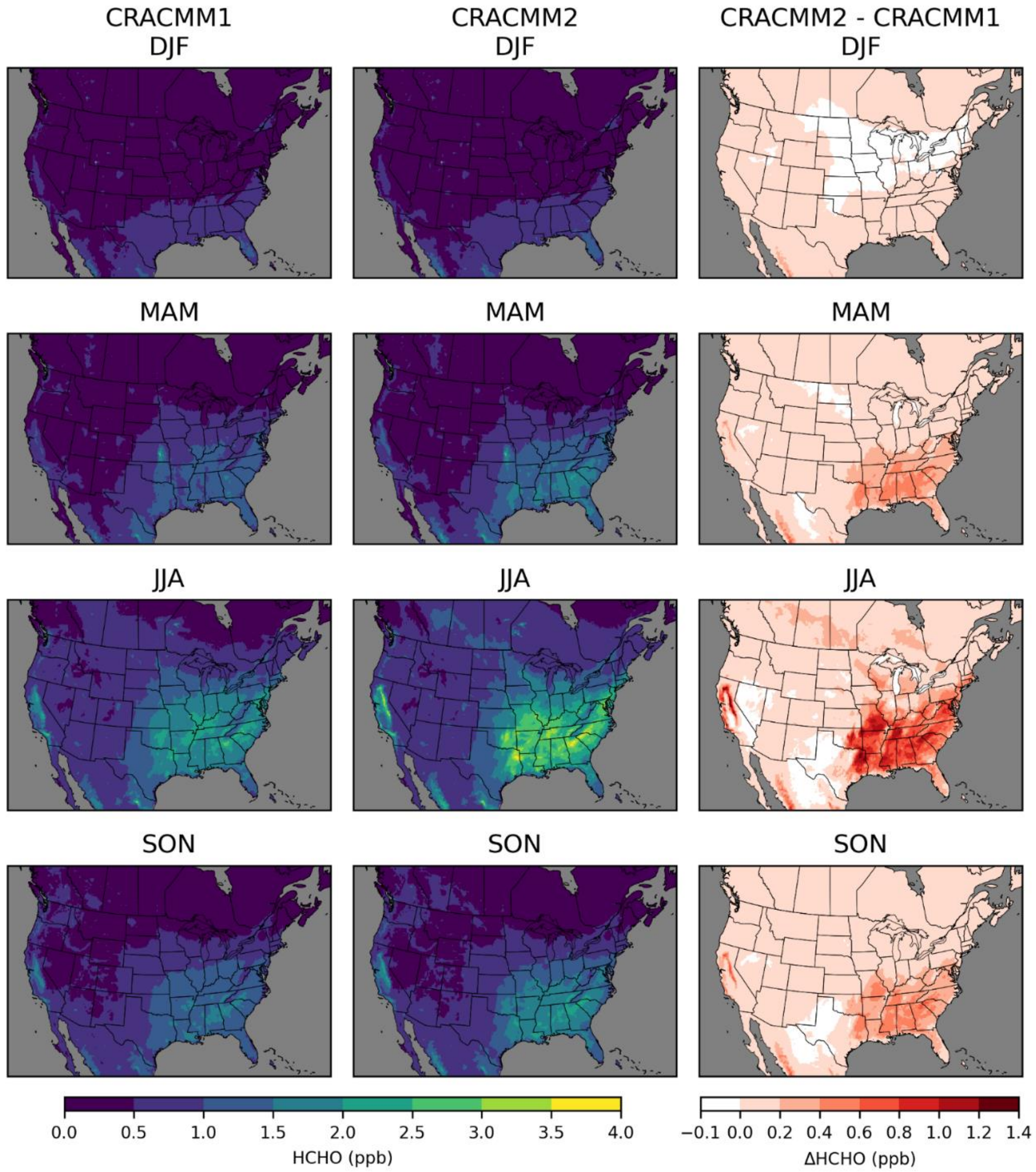

210 **Figure S9. Surface layer 2019 seasonal average HCHO concentrations averaged over 11 am–3 pm local time simulated with CRACMM1 (left column) and CRACMM2 (middle column) and the change in CRACMM2 compared to CRACMM1 (right column).**

The effects of adding ECH4 to CRACMM2 are mostly localized in areas with extremely high ECH4 in the emissions inventory (Fig. S10). The largest impacts are seen in northeastern Wyoming and around the border between New Mexico and Colorado. Impacts are also seen in Alberta, Canada, where there is substantial oil and gas activity. Small impacts from ECH4 can also be seen in the Central Valley of California and in southern California. ECH4 is small compared to the fixed CMAQ global background methane concentration of 1850 ppb, so we do not expect to see significant effects on HCHO from ECH4 other than from extremely large sources. ECH4 as it is represented in the emissions inventory also does not provide a full accounting of methane emissions. Not all sources of methane are inventoried in the NEI (e.g., waterbodies). Effects of emitted methane will therefore not be captured for the sources that are not traditionally included in the emissions inventory. Impacts on HCHO from adding heterogeneous uptake of HO_2 and nitrate are small and affect mostly the southeastern US. There are two likely contributing factors. One is decreased HO_X from uptake of HO_2 marginally increasing the lifetime of HCHO. The other is a decrease in the favorability of the RO_2+HO_2 channel with reduced HO_2 and resulting increase in the favorability of the RO_2+NO channel which has higher HCHO yields compared to the RO_2+HO_2 route. The addition of new heterogeneous uptake pathways was not aimed at improving HCHO but were opportunistic updates targeted for implementation in CRACMM2.

The impacts on HCHO from the updates to aromatic chemistry are small and extremely localized compared to some of the other updates. These effects come from the newly added explicit styrene species (STY) in CRACMM2. Most styrene in the emissions inventory (~55%) comes from non-EGU point sources which are mostly made up of industrial sources. During the typical peak of photochemistry from 11am-3pm, the largest impacts are seen in the Los Angeles, California, area and along the border with Mexico near Tijuana. Impacts can also be seen around the Great Lakes region, particularly near Chicago, Illinois, and near the Puget Sound in Washington. More impacted areas can be seen if we look instead at the change in HCHO over all hours rather than focusing only on the peak of photochemistry. The other updates to aromatics do not change any chemistry. They only involve changes in how emissions are mapped, and these changes have little impact on HCHO. Although the effects on HCHO from the aromatic chemistry updates are relatively small, there is additional value in the addition of two new HAPs, ethylbenzene (EBZ) and styrene (STY), which are now represented explicitly in CRACMM2.

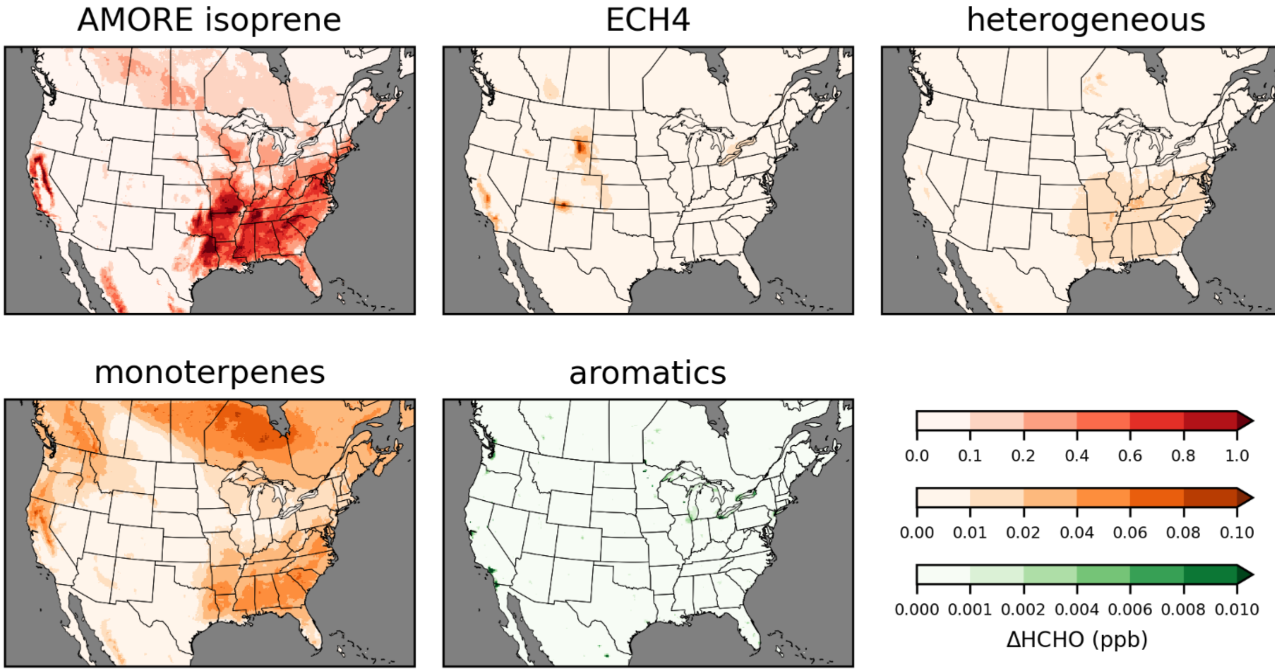

Figure S10. Incremental impacts on surface layer 11 am–3 pm local time 2019 June–August average HCHO concentration resulting from chemistry updates. Color bar scales for incremental impacts differ depending on the magnitude of the impacts. The red color scale is used for the AMORE isoprene impacts; the orange color scale is used for the ECH4, heterogeneous uptake, and monoterpene impacts; the green color scale is used for aromatic impacts.

Additional TROPOMI results

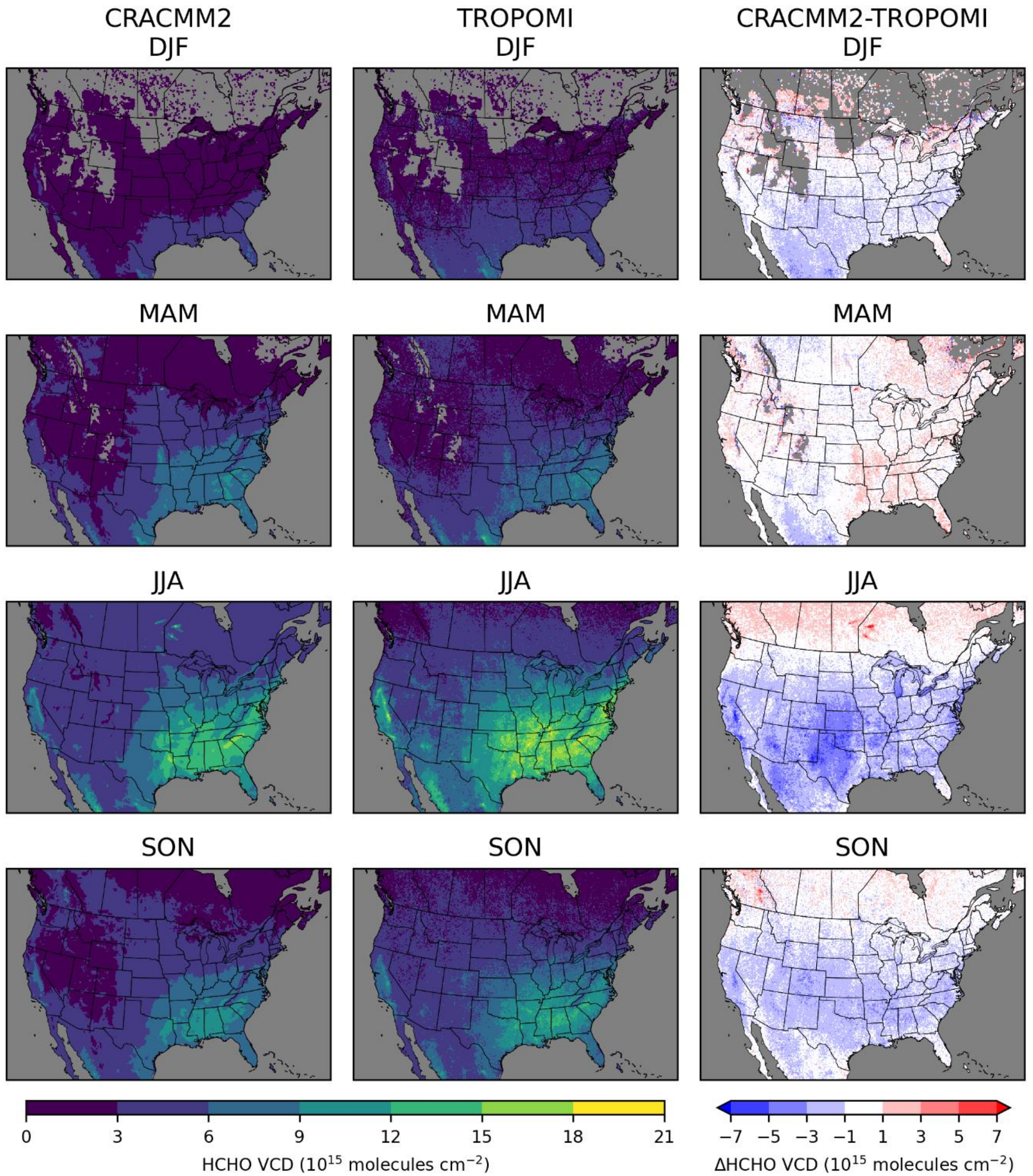

Figure S11. 2019 seasonal average tropospheric vertical column densities from CMAQ with CRACMM2 (left) and from TROPOMI (middle), and the difference between CRACMM2 and TROPOMI (right).

We incorporate an uncertainty estimate into the comparison of HCHO VCD from CMAQ with CRACMM2 to TROPOMI using uncertainty estimates provided in the TROPOMI HCHO algorithm theoretical basis document (ATBD) (KNMI, 2022). Table 13 of the ATBD reports uncertainties in monthly averaged columns for low (<=1×10^{16} molecules cm^{-2}) and elevated (>1×10^{16} molecules cm^{-2}) columns as 50% and 25%, respectively, as the total uncertainty neglecting uncertainties in the AMF. We neglect the uncertainties in the AMF since we have incorporated information from the TROPOMI averaging kernel into the CMAQ VCD which is the suggested approach in the ATBD. The uncertainties for low and elevated columns are applied to the June-August 2019 seasonal averages to calculate lower and upper bounds for TROPOMI HCHO VCD which are compared to CRACMM2 along with the original (i.e., without uncertainty estimate) TROPOMI HCHO VCD (Fig. S12). The HCHO VCD in CMAQ with CRACMM2 is mostly within the limits of uncertainty of the TROPOMI HCHO VCD. Notable exceptions to this are in the Permian Basin; near Phoenix, Arizona; Los Angeles, California; and other parts of California east of the Central Valley.

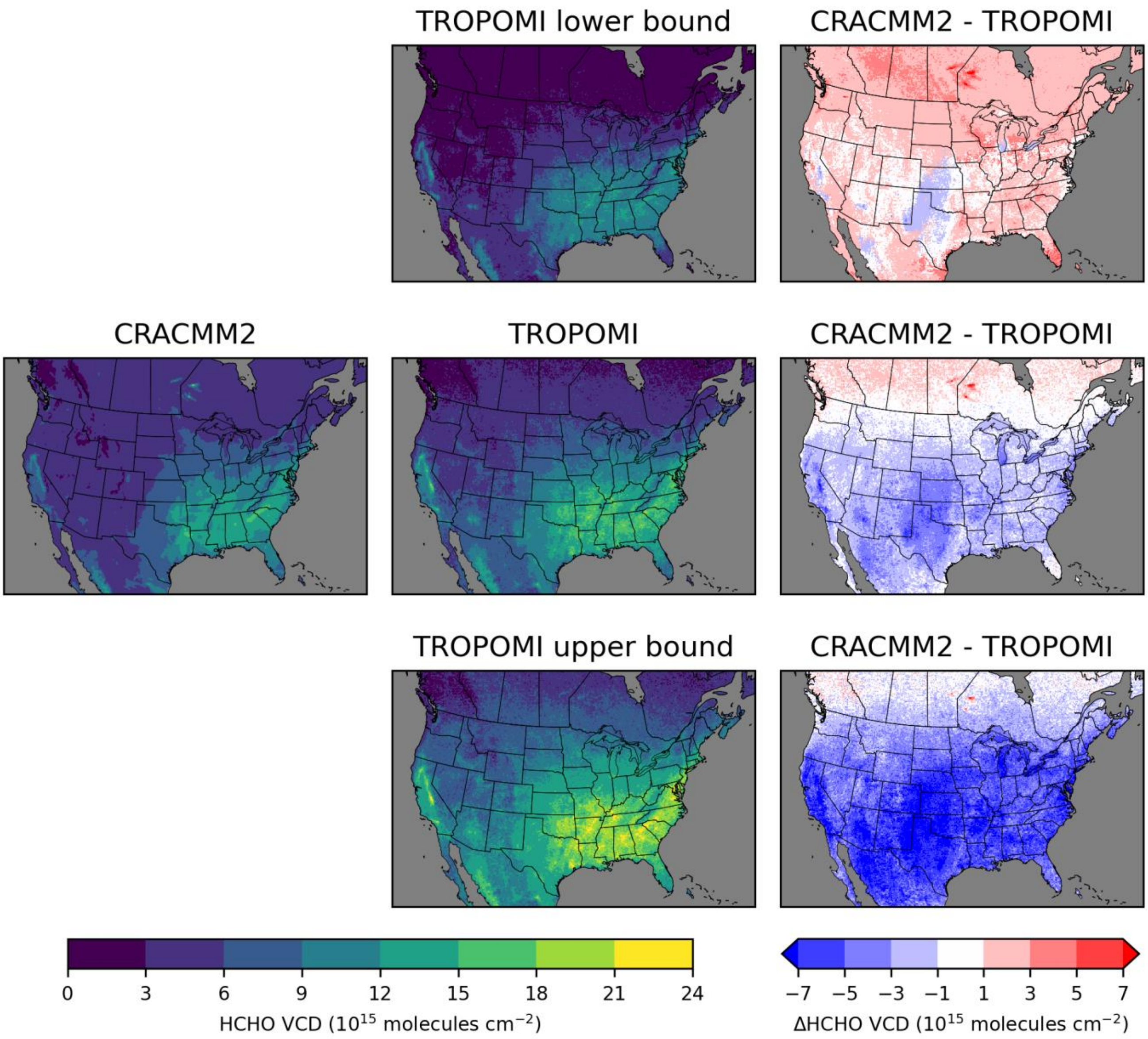

Figure S12. 2019 June-August average tropospheric vertical column densities from CMAQ with CRACMM2 (left column), TROPOMI with CRACMM2-based AMF (middle column), and the difference between CRACMM2 and TROPOMI (right column). The top row uses the lower bound for TROPOMI based on the uncertainty analysis. The middle row does not incorporate uncertainty. The bottom row uses the upper bound for TROPOMI based on the uncertainty analysis.

Oil and gas emission sensitivity simulation results

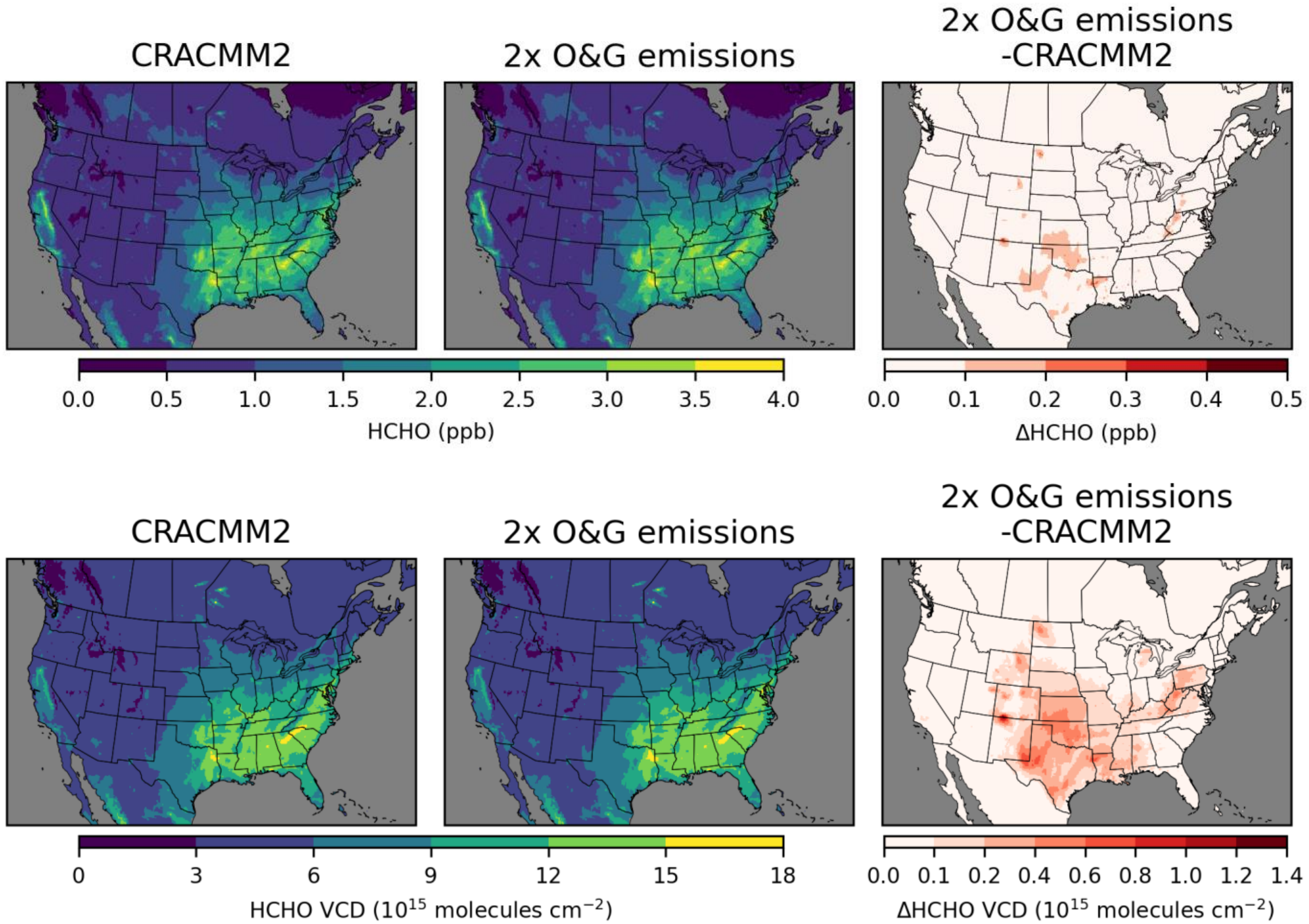

Figure S13. Average June-August 2019 change in HCHO in CMAQ at the surface from 11am-3pm (top row) and in the vertical column at the TROPOMI overpass time (~1:30pm local solar time) from doubling oil and gas NO_X and ROC emissions.

HCHO AQS evaluation

HCHO observations are available from the Air Quality System (AQS) database which contains data from federal, state, local, and tribal air quality monitoring stations. Routine HCHO measurements are primarily taken at sites in the Photochemical Assessment Monitoring Stations (PAMS) and National Air Toxics Trends Sites (NATTS) networks. HCHO is measured using method TO-11A in which HCHO is collected on 2,4-dinitrophenylhydrazine (DNPH) coated cartridges from which HCHO derivative products are measured offline using high performance liquid chromatography (HPLC). HCHO data shown here are obtained from the AQS website (https://aqs.epa.gov/aqsweb/airdata/download_files.html) and have not been corrected for field blanks; however, the values of field blanks are expected to be small enough that any qualitative conclusions are unaffected. PAMS and NATTS sites typically have sample collection schedules of three 8-h samples or one 24-h sample which limits the usefulness for evaluation of the diurnal variability of HCHO. The DNPH measurement technique has also recently been found to measure lower HCHO values compared to other instruments in Atlanta, GA, (Mouat et al., 2024) and in Salt Lake City, UT, (Jaffe et al., 2024). While there is some uncertainty in these measurements, we report a comparison between the daily average HCHO observations available in AQS and daily averages from CMAQ using CRACMM1 and CRACMM2 (Figure S14). A total of 5424 daily average observations are available from a total of 109 sites in 2019. HCHO is underestimated, but the bias is improved by about 15%. There are, however, a limited number of sites in the southeastern US (Figure S15) where HCHO in CRACMM2 increased the most which limits the usefulness of the comparisons to AQS data in evaluating the CRACMM2 updates.

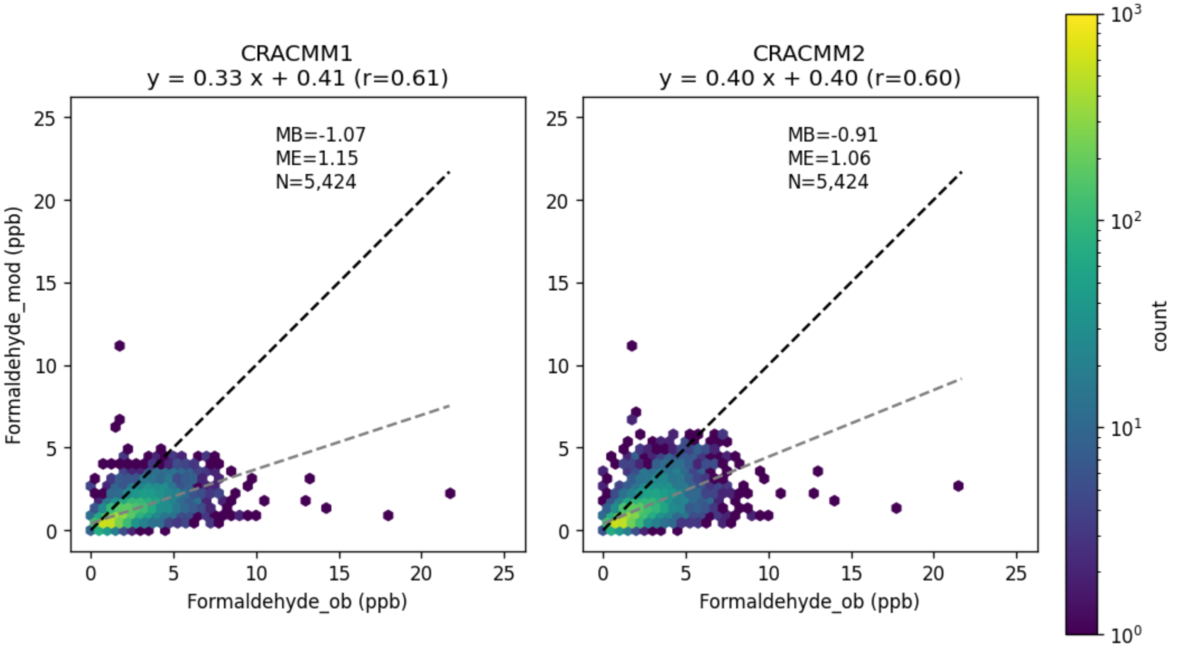

Figure S14. Density scatter plots of daily average HCHO observations from AQS compared to simulated daily averages from CMAQ using CRACMM1 (left) and CRACMM2 (right).

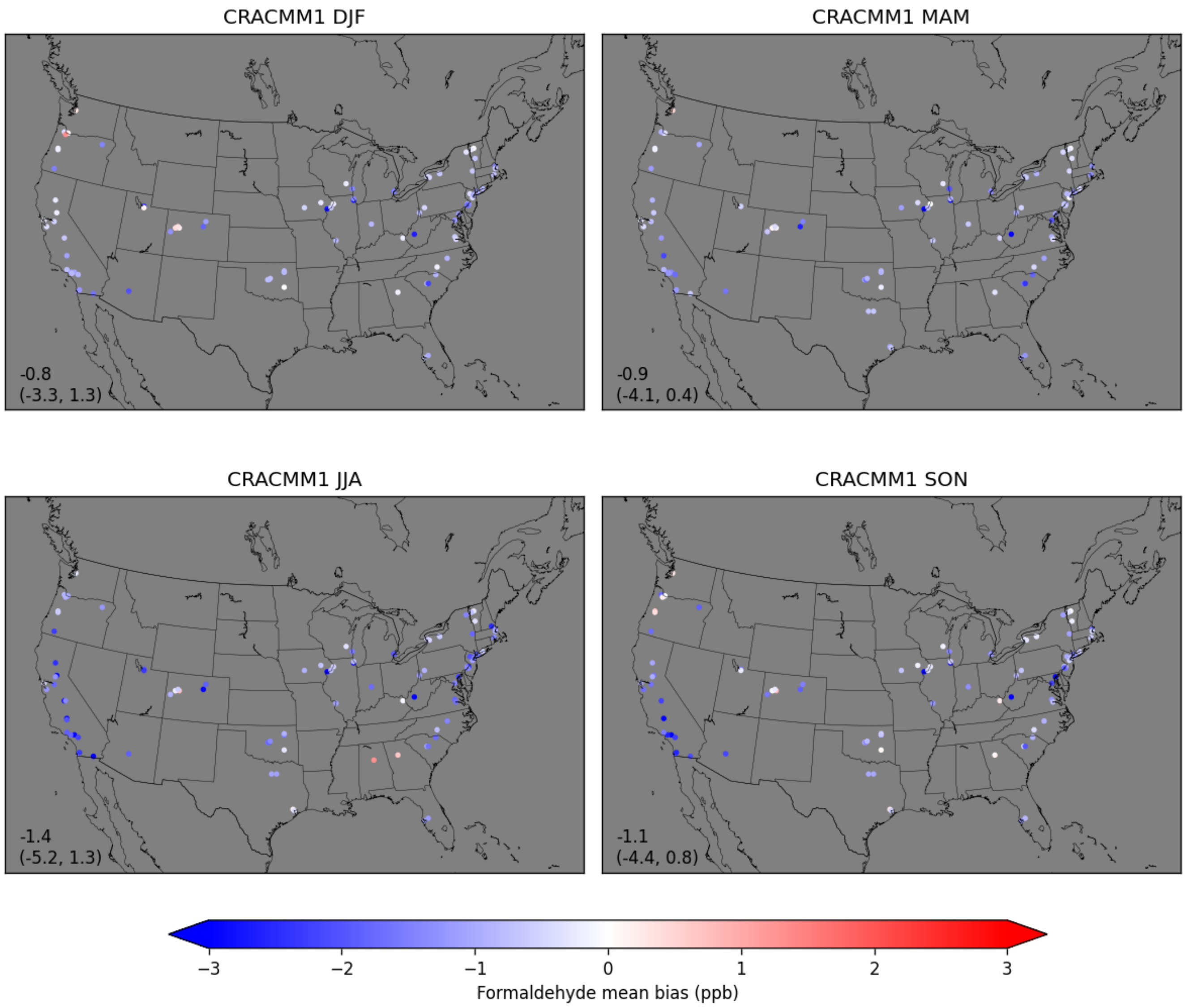

Figure S15. Seasonal average bias in daily average HCHO with CRACMM1 compared to observed HCHO from AQS sites. Annotations in the lower left of each panel show the mean (min, max) of seasonal average biases over all sites.

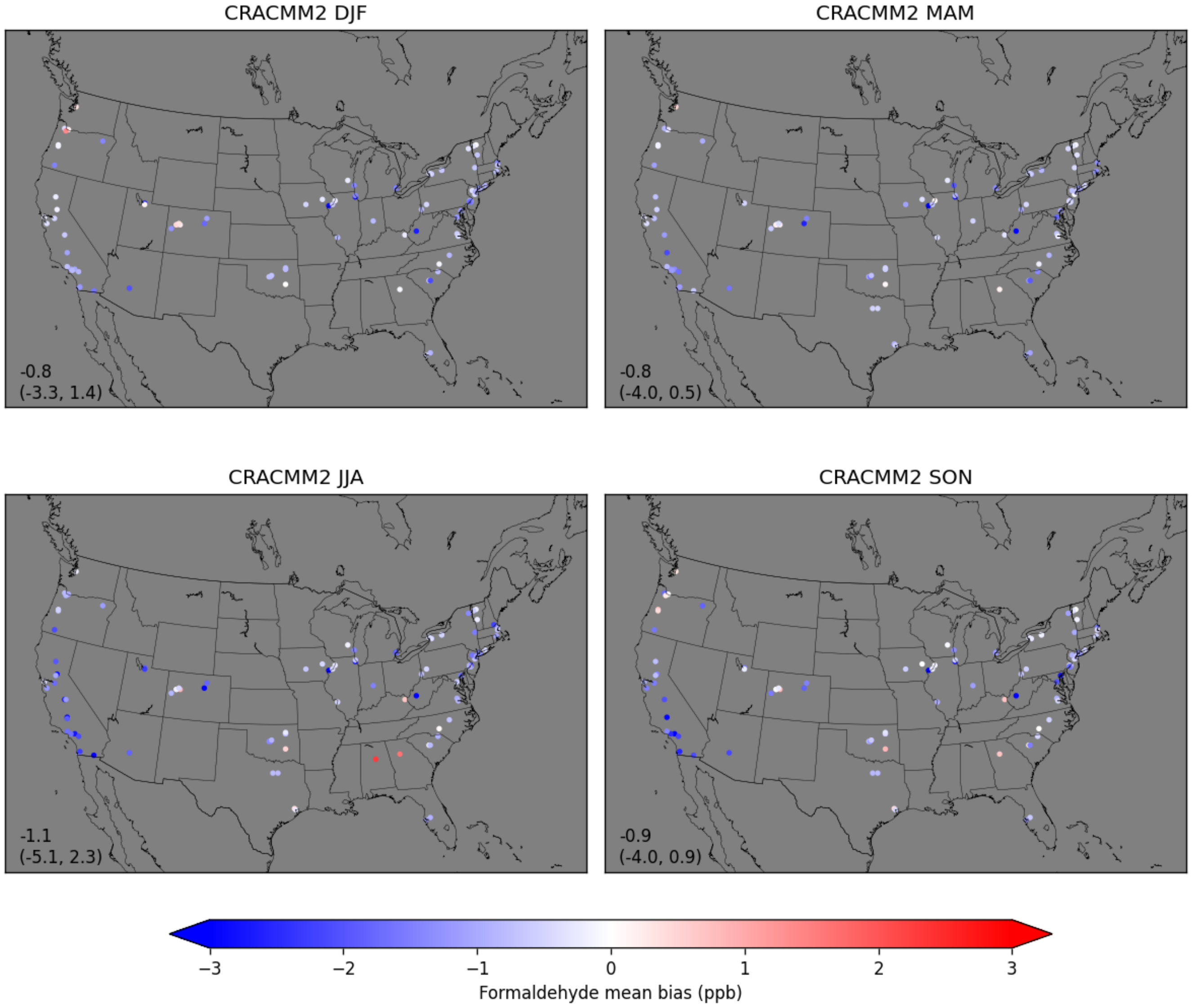

Figure S16. Seasonal average bias in daily average HCHO with CRACMM2 compared to observed HCHO from AQS sites. Annotations in the lower left of each panel show the mean (min, max) of seasonal average biases over all sites.

305

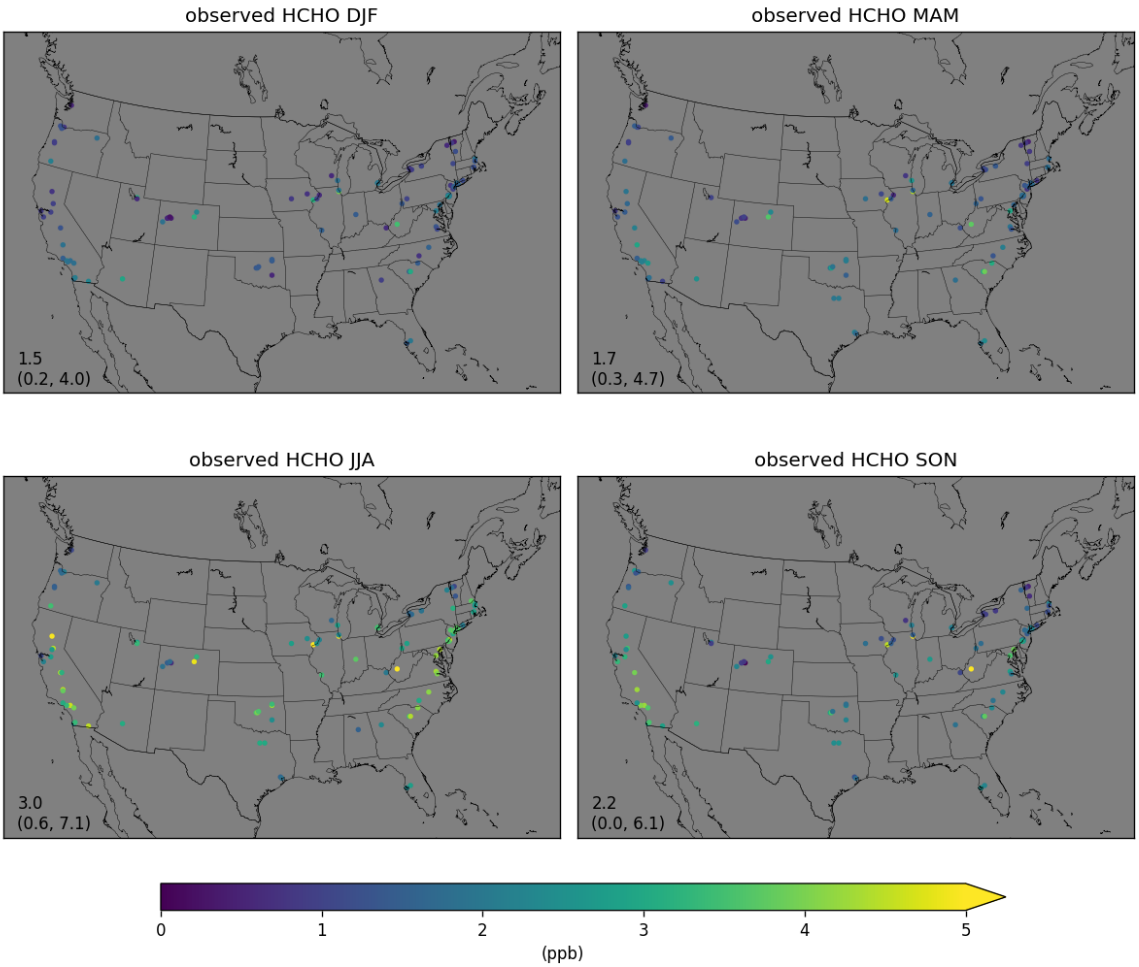

Figure S17. Seasonal average observed daily average HCHO from AQS sites. Annotations in the lower left of each panel show the mean (min, max) of seasonal average observations over all sites.

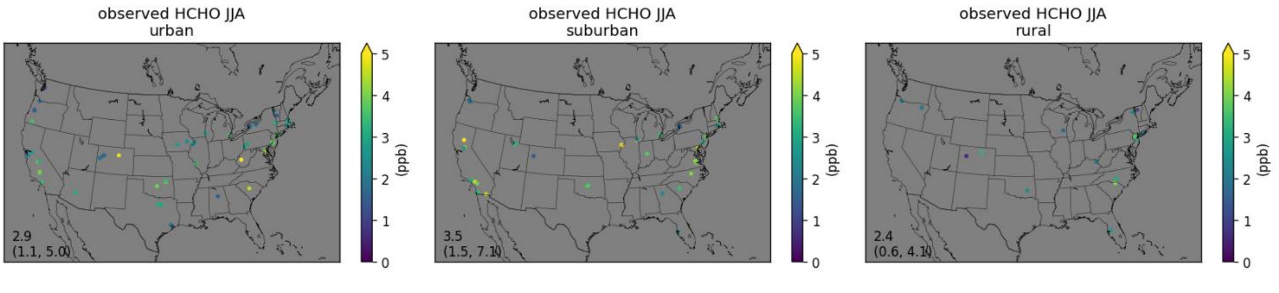

Figure S18. June-August 2019 average observed daily average HCHO from AQS sites, separated by their location in urban, suburban, and rural areas. Annotations in the lower left of each panel show the mean (min, max) of seasonal average observations over all sites.

Hourly observations

Table S4. Hourly HCHO measurement locations, sampling dates, and instrumentation.

Location	Sampling dates	Instrument	Uncertainty	Additional notes
Westport, CT	2023 May 22 – Nov 14	Aerodyne TILDAS	Maximum of 0.6 ppb or 15%	Collected in support of 2023 Synergistic TEMPO Air Quality Science (STAQS). Available from https://www-air.larc.nasa.gov/cgi-bin/ArcView/listos.2023?GROUND-WESTPORT=1
	2018 Jun 21 – Sep 4	Aerodyne TILDAS	10%	Collected during the 2018 Long Island Sound Tropospheric Ozone Study (LISTOS). Available from https://www-air.larc.nasa.gov/cgi-bin/ArcView/listos?GROUND-WESTPORT=1
Flax Pond, NY	2023 May 22 – Nov 16	Aerodyne TILDAS	Maximum of 0.6 ppb or 15%	Collected in support of 2023 STAQS. Available from https://www-air.larc.nasa.gov/cgi-bin/ArcView/listos.2023?GROUND-FLAX-POND=1
New Brunswick, NJ	2023 May 22 – Nov 8	Picarro G2307	Maximum of 0.6 ppb or 15%	Collected in support of 2023 STAQS. Available from https://www-air.larc.nasa.gov/cgi-bin/ArcView/listos.2023?GROUND-RUTGERS=1
Sheboygan, WI	2017 Jun 26 – Jul 22	Aerodyne TILDAS	10%	Collected during the 2017 Lake Michigan Ozone Study (LMOS). Available from https://www-air.larc.nasa.gov/cgi-bin/ArcView/lmos?GROUND-SHEBOYGAN=1
Salt Lake City, UT	2017 Jan 15 – Feb 14	Aerodyne TILDAS	10%	Collected during the 2017 Utah Winter Fine Particle Study (UWFPS). Available from https://csl.noaa.gov/groups/csl7/measurements/2017uwfps/Ground/DataDownload/index.php?page=/groups/csl7/measurements/2017uwfps/Ground/DataDownload/
Research Triangle Park, NC	2016 Aug 16 – Dec 11	Aerodyne TILDAS	10%	Collected near EPA campus
Atlanta, GA (South DeKalb)	2022 Apr 26 – 2023 Oct 31	Picarro G2307	10%	See Mouat et al., 2024
Atlanta, GA (Georgia Tech)	2022 Jul 25 – 2023 Jan 31	MIRA Ultra	14% + 0.3 ppb	See Mouat et al., 2024

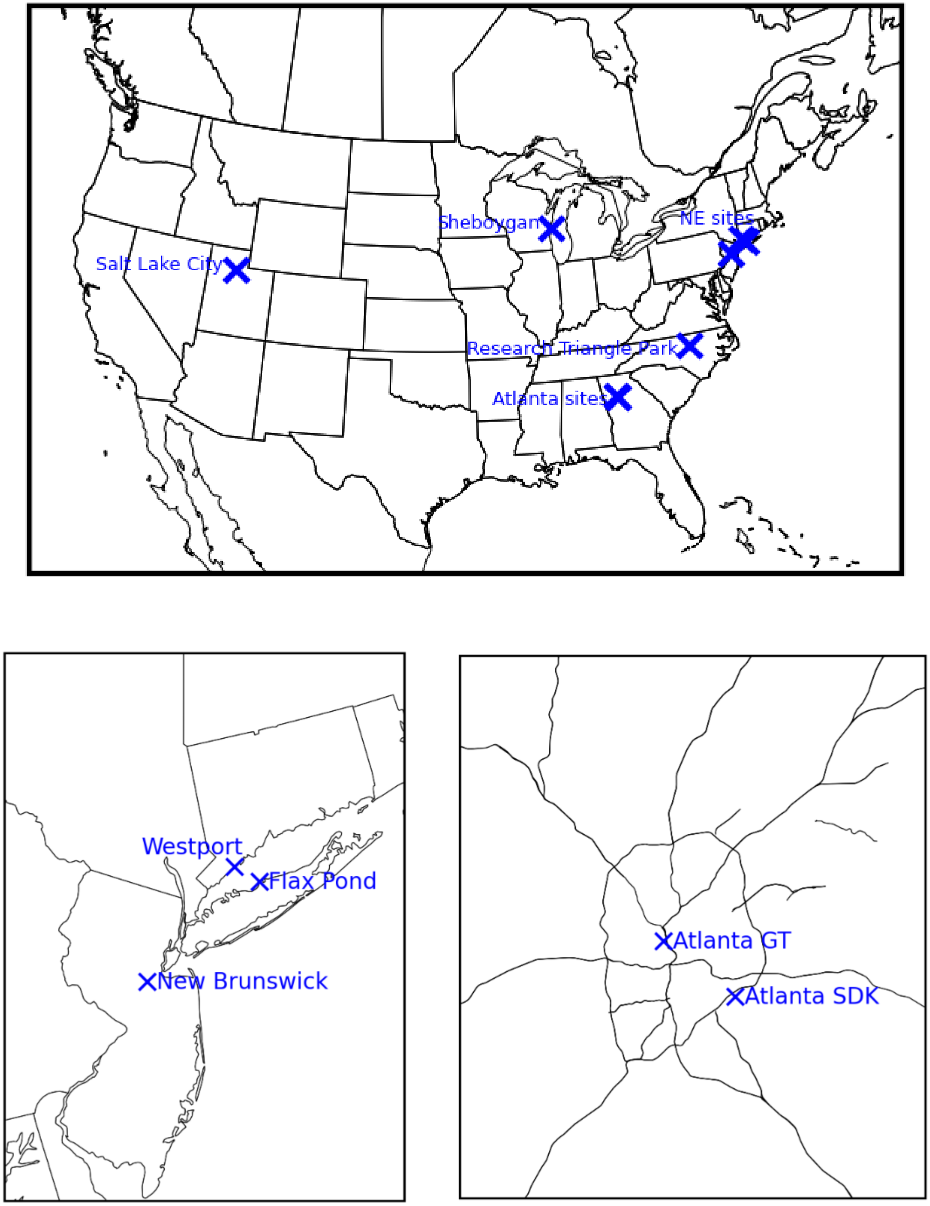

320 **Figure S19. Map of sampling locations listed in Table S4. The two maps in the bottom row show a zoomed in view of the northeastern (NE) US sites and the Atlanta sites. On the map for the Atlanta sites, major roadways are displayed to provide additional geographic context.**

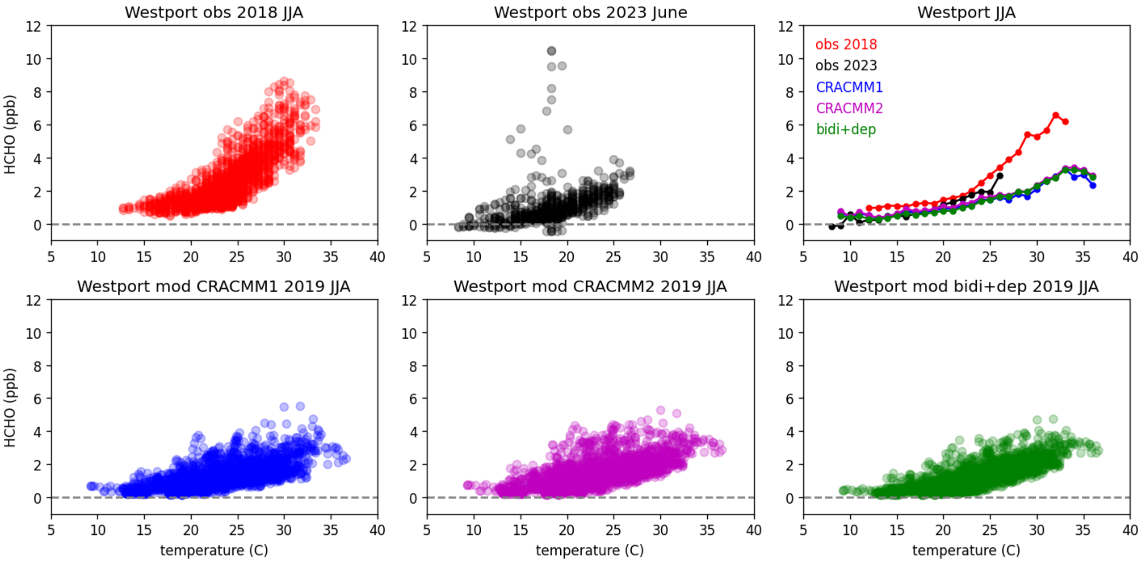

Figure S20. Hourly HCHO concentration by temperature at the Westport, CT, sampling location during summer. All available hourly data points are provided for the 2018 and 2023 observations and for the CRACMM1, CRACMM2, and bidi+dep CMAQ simulations. The upper right panel shows the median temperature in 1 degree C temperature bins for each set of observations and modeled concentrations.

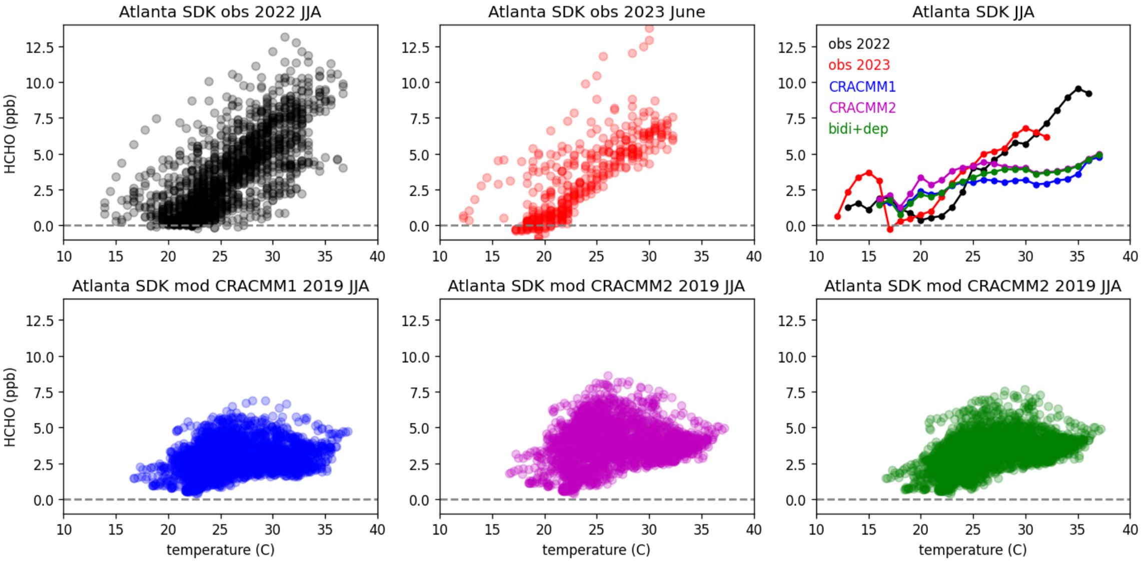

Figure S21. Hourly HCHO concentration by temperature at the Atlanta, GA South DeKalb (SDK) sampling location during summer. All available hourly data points are provided for the 2022 and 2023 observations and for the CRACMM1, CRACMM2, and bidi+dep CMAQ simulations. The upper right panel shows the median temperature in 1 degree C temperature bins for each set of observations and modeled concentrations.

Observations from fall 2016 in Research Triangle Park, NC, show a rapid rise in the early morning leading to a midday peak which is not captured in the CMAQ simulations. The CRACMM2 updates do increase the HCHO at midday (as well as throughout the entire day) which makes the simulated values closer to the observed at midday but results in higher HCHO at night. The diurnal profile of the CMAQ simulations is much flatter than is seen in the observations. The other observations included in Figure S22 are during summer. For the Sheboygan, WI, data the diurnal profile is mostly flat with small late morning and evening peaks. The peak values simulated with CRACMM2 are close to those seen in the observations, despite the shape of the diurnal profile not aligning well with the observations. We note also that this sampling location is on the shore of Lake Michigan which has complex land-sea breeze effects which are not always well simulated, so meteorology could play an important role here. The observations for Westport, CT, during 2018 are similar to the 2023 observations at the same site with HCHO beginning to rise in the early morning, peaking around noon, and then falling as the afternoon progresses and into night. The major difference between the 2018 and 2023 data is the higher midday peak in the 2018 observations. The CMAQ data shown for Westport, CT, below are identical to what is shown in Figure 6 for the Westport 2023 data.

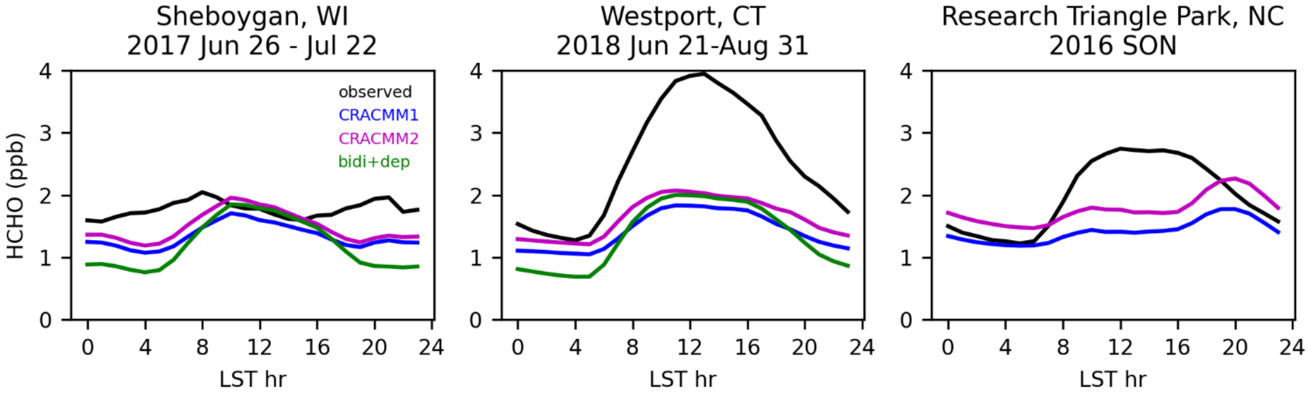

Figure S22. Diurnal profiles of observations in several years at several sites compared to CMAQ simulations in 2019 using CRACMM1, CRACMM2, and CRACMM2 with updated HCHO bidirectional flux and deposition (bidi+dep). The bidi+dep results are not shown for the Research Triangle Park, NC, site since this was a sensitivity simulation conducted for summer only. Sampling locations and dates are provided above each panel.

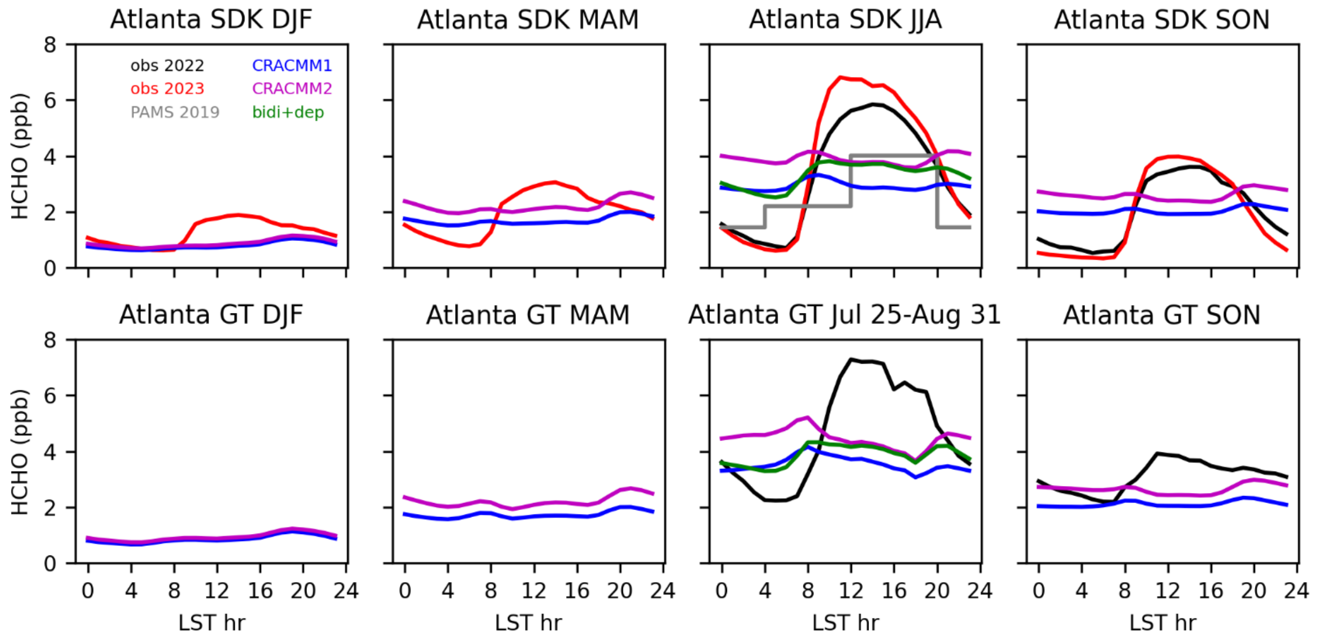

Figure S23. Seasonal average diurnal variability at South DeKalb (SDK) and Georgia Tech (GT) sites.

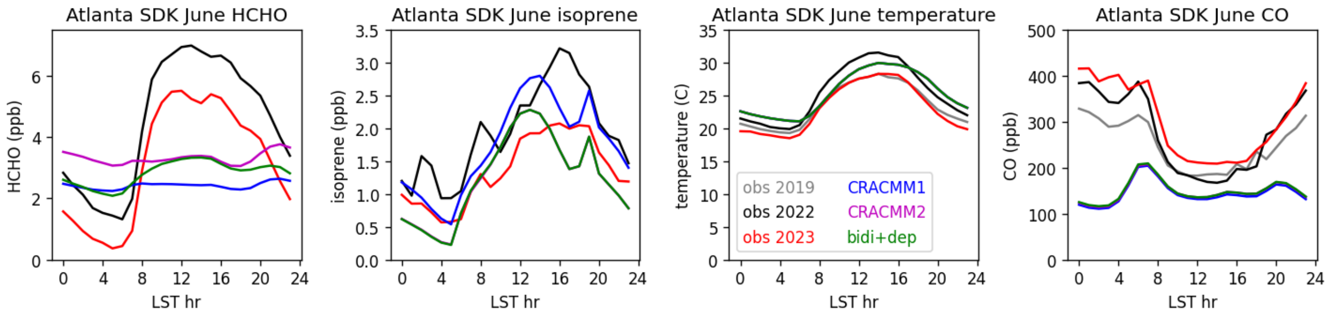

Figure S24. Hourly HCHO, isoprene, temperature, and CO during June for various years at Atlanta SDK PAMS site.

Effects of HCHO bidirectional flux and deposition updates

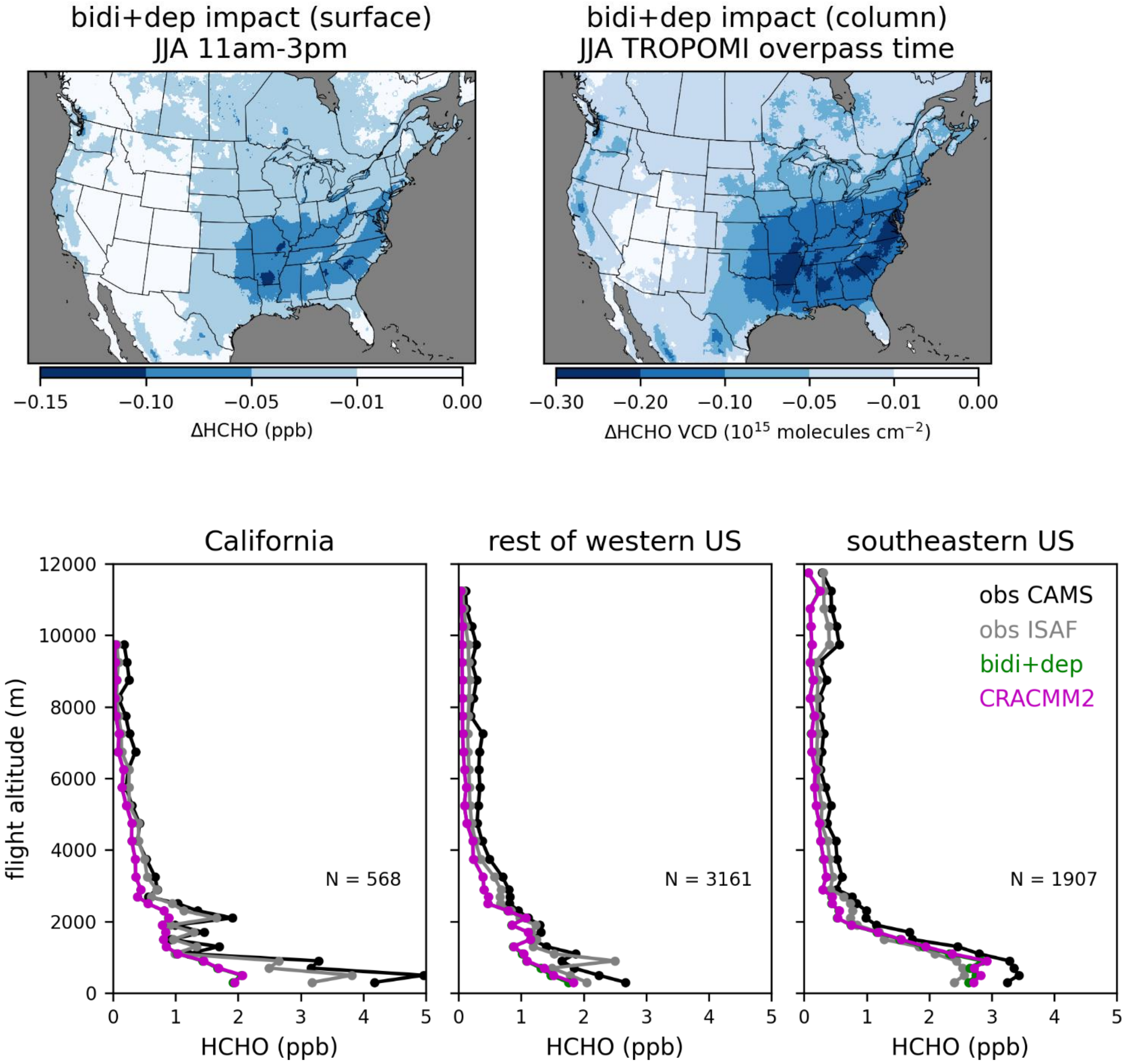

Figure S25. Effects of bidirectional flux and deposition updates on HCHO for 2019 June-August 11am-3pm surface HCHO (top left), 2019 June-August average HCHO VCD at the TROPOMI overpass time (top right), and on the HCHO vertical profile comparison to FIREX (bottom).

Seasonal average HCHO controllable fractions

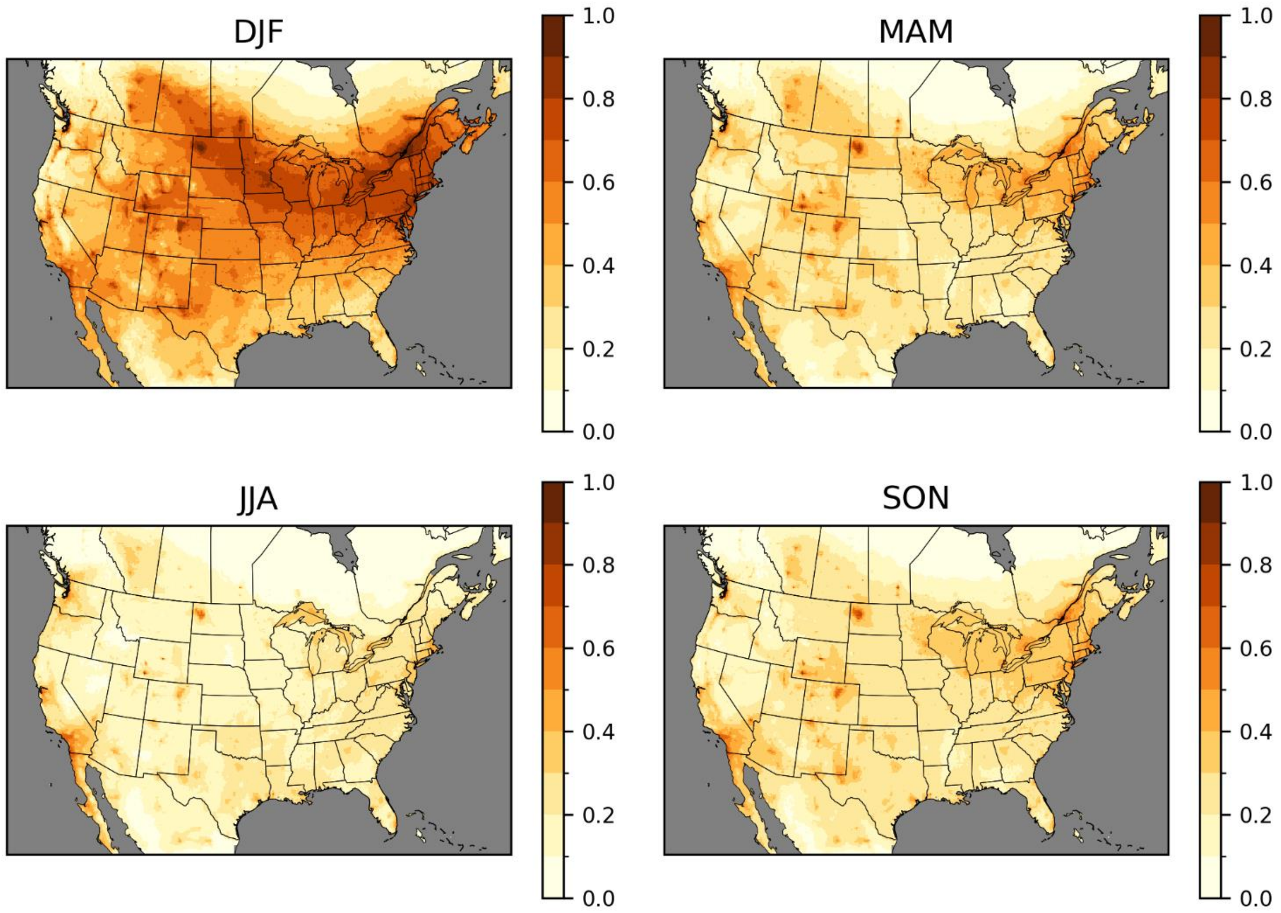

Figure S26. Seasonal average controllable fraction of HCHO calculated as the difference between the base simulation and the zero US anthropogenic NOx and ROC simulation divided by the base simulation.

CRACMM2 ozone evaluation

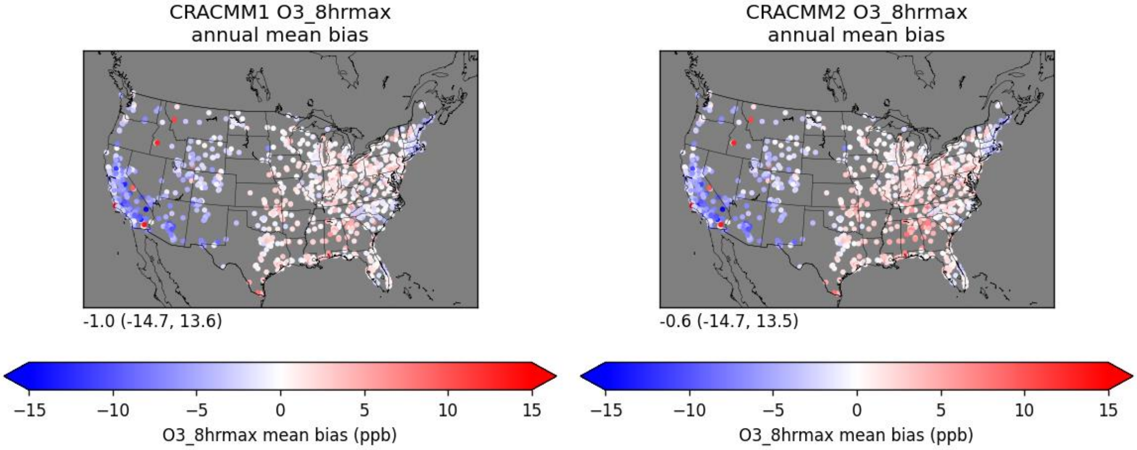

Figure S27. Annual mean bias in MDA8 O_3 for 2019 at AQS monitoring sites for CRACMM1 (left) and CRACMM2 (right). The annotations in the lower left show the mean (min, max) site average biases.

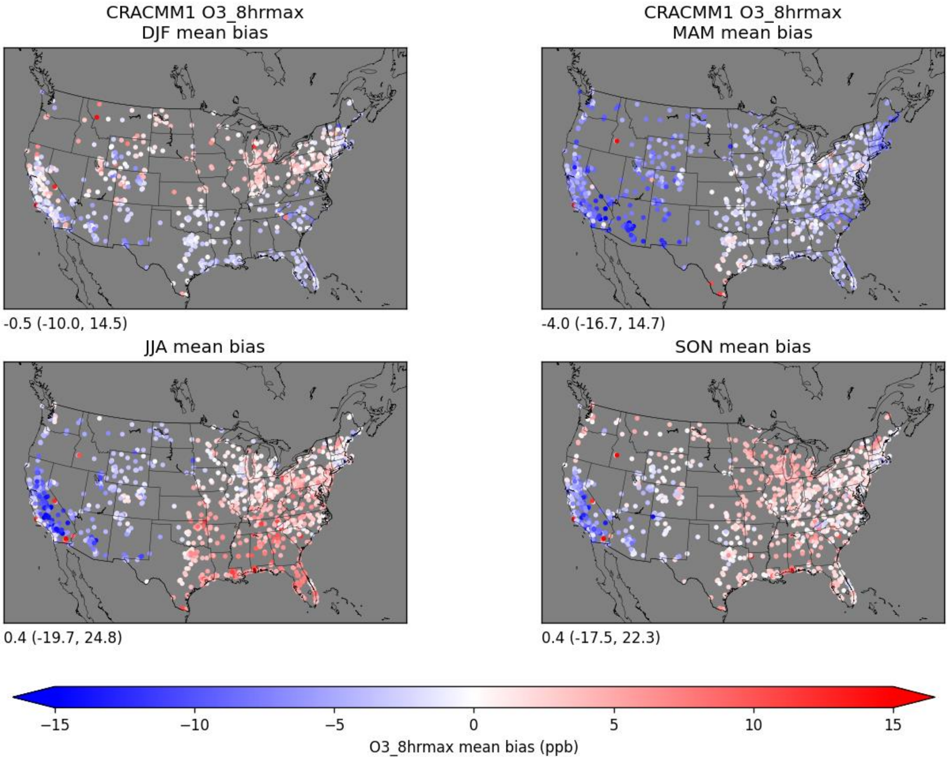

Figure S28. Seasonal mean bias in MDA8 O_3 for 2019 at AQS monitoring sites for CRACMM1. The annotations in the lower left show the mean (min, max) site average biases.

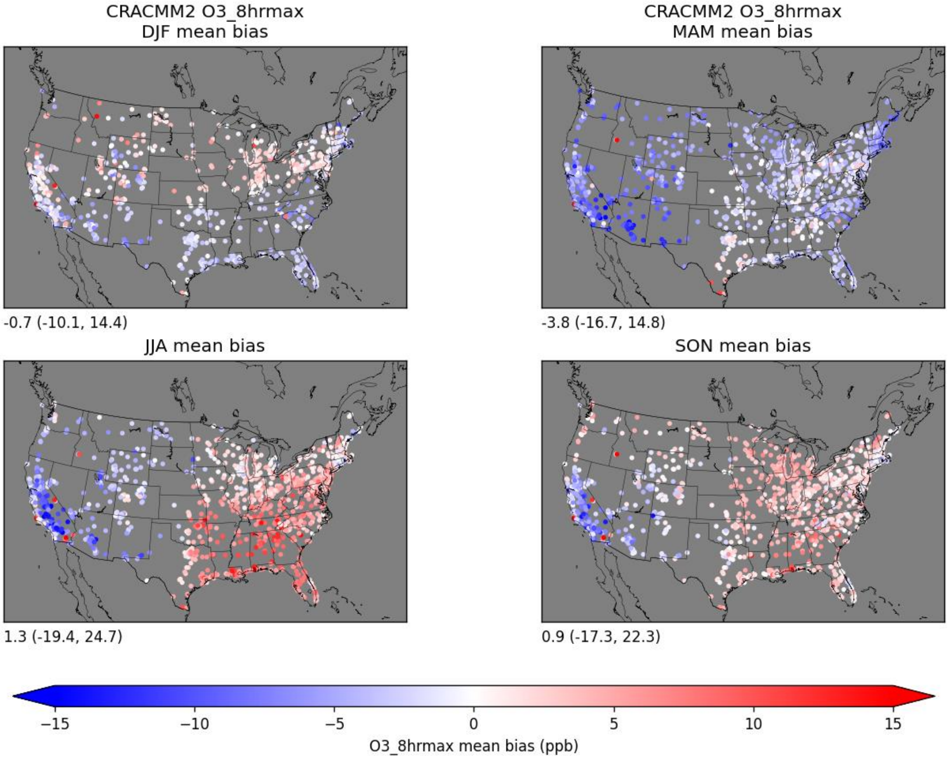

Figure S29. Seasonal mean bias in MDA8 O_3 for 2019 at AQS monitoring sites for CRACMM2. The annotations in the lower left show the mean (min, max) site average biases.

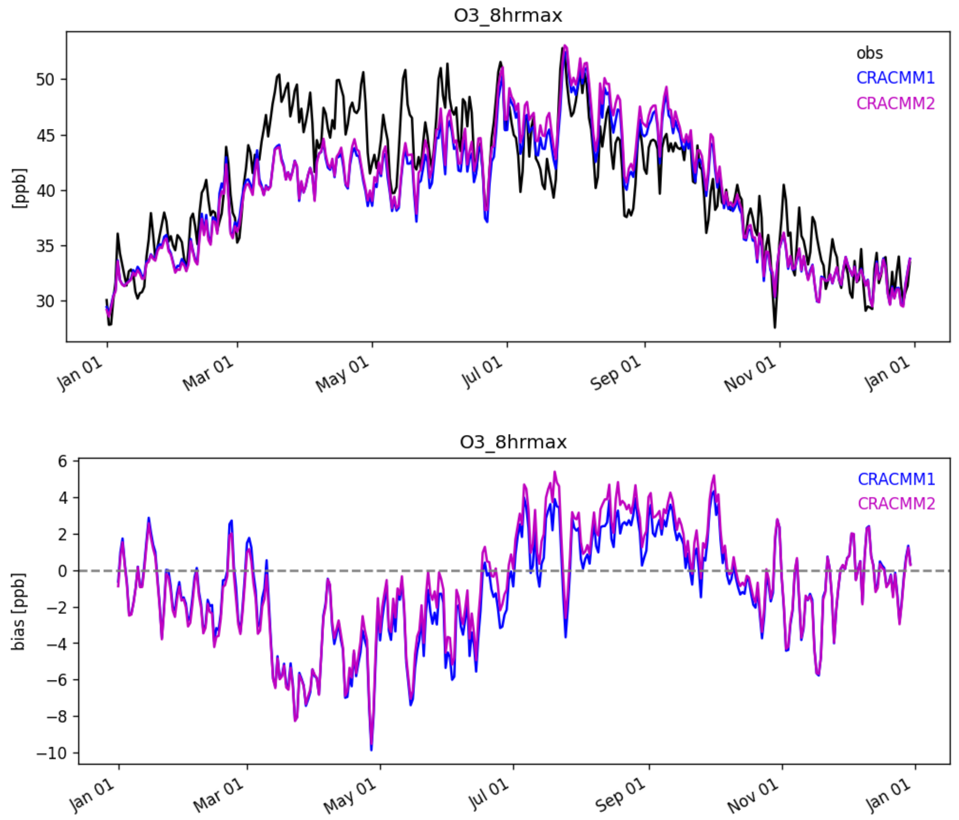

Figure S30. Daily average MDA8 O_3 (top) and bias (bottom) for 2019 averaged over all AQS monitoring sites for CRACMM1 and CRACMM2.

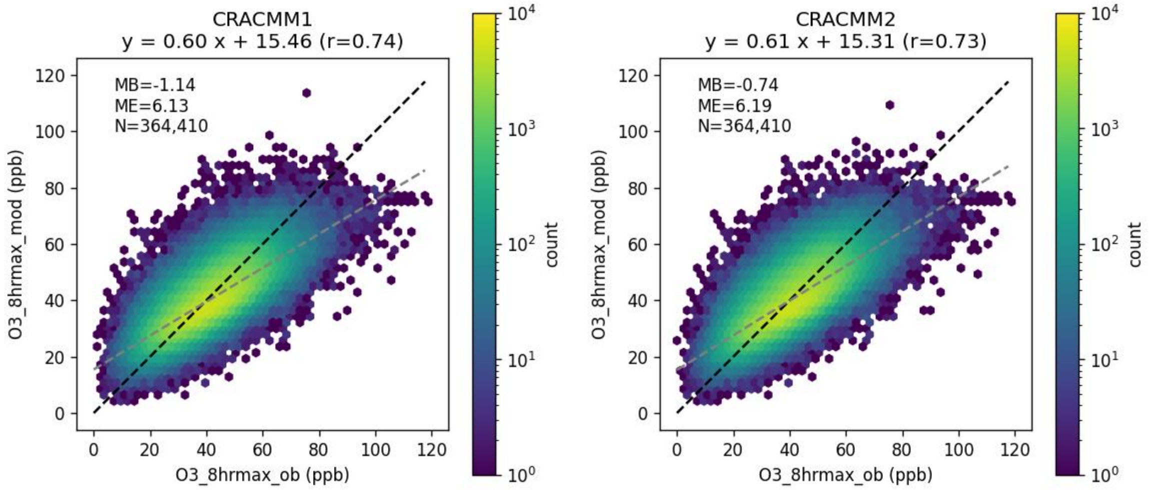

Figure S31. Density scatter plot of observed MDA8 O_3 vs. CRACMM1 (left) and CRACMM2 (right). The mean bias (MB), mean error (ME), and number of observations (N) are indicated on each panel.

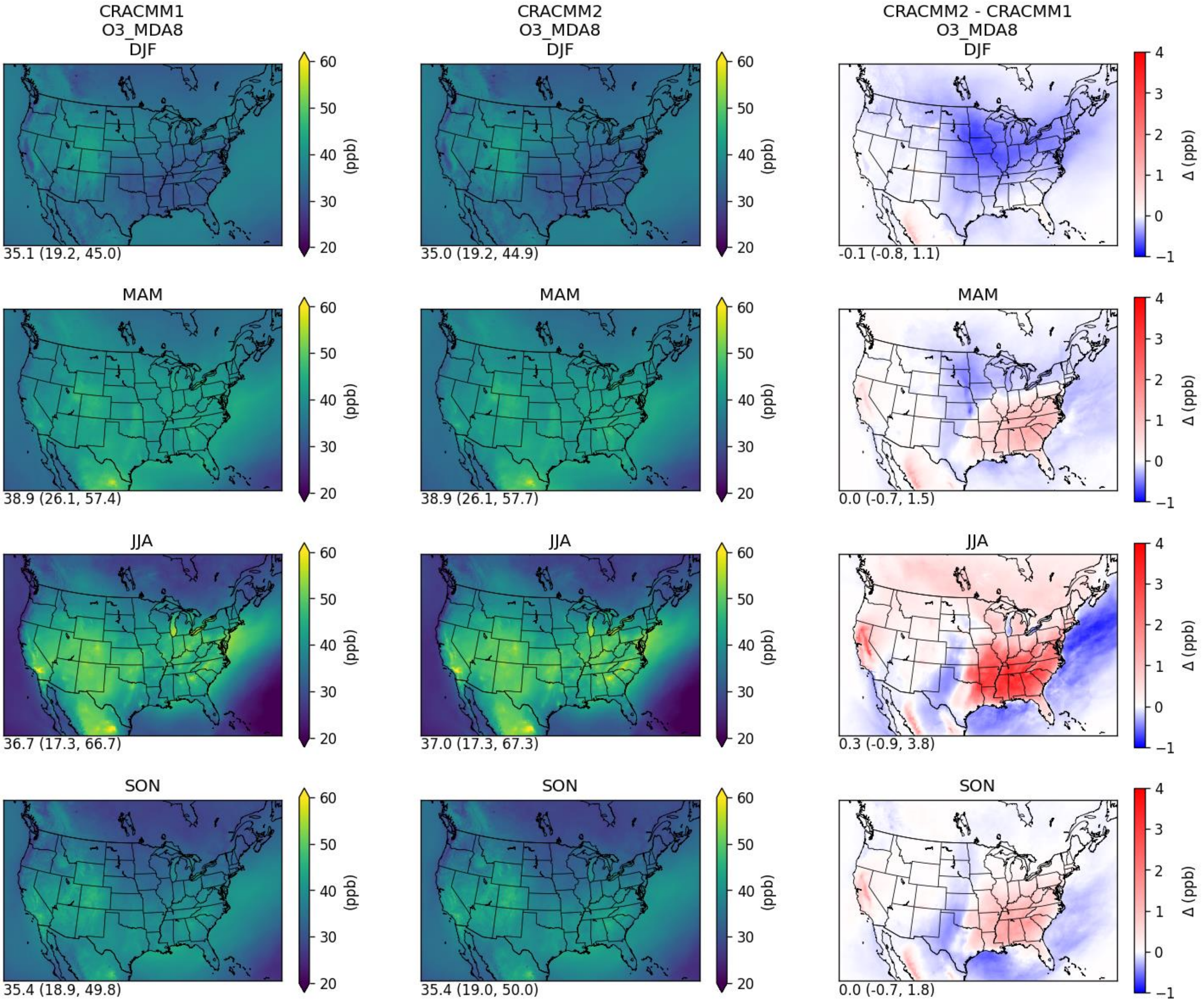

Figure S32. Seasonal average MDA8 O_3 in CRACMM1 (left column) and CRACMM2 (middle column) and the change in CRACMM2 compared to CRACMM1 (right column). The annotations in the lower left show the mean (min, max) over the modeling domain.

CRACMM2 PM2.5 evaluation

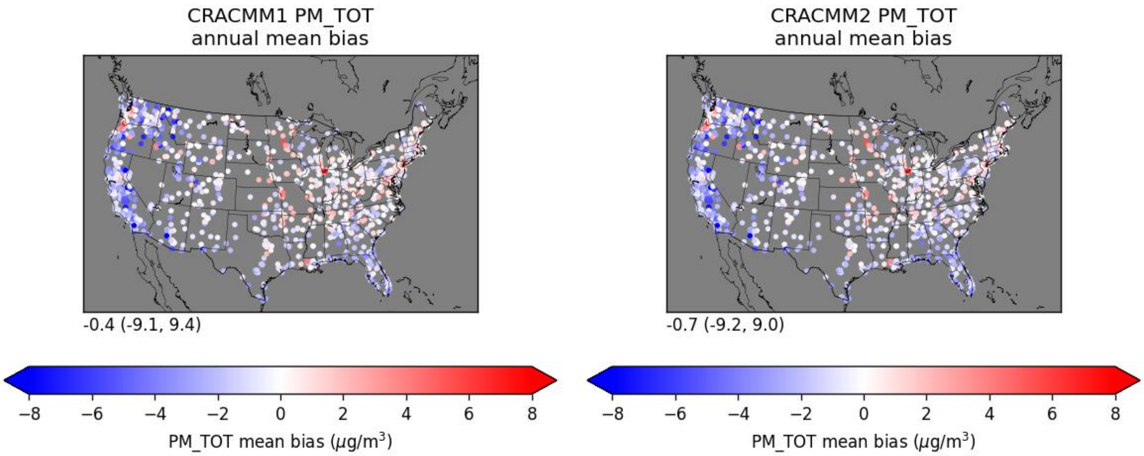

Figure S33. Annual mean bias in daily average $PM_{2.5}$ for 2019 at AQS monitoring sites for CRACMM1 (left) and CRACMM2 (right). The annotations in the lower left show the mean (min, max) site average biases.

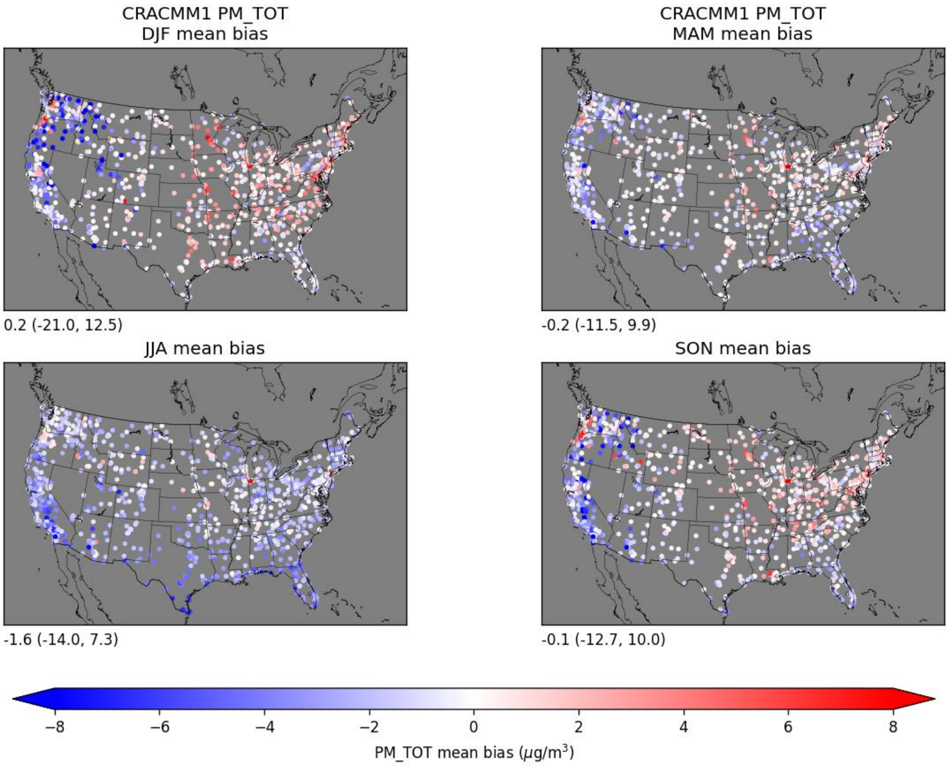

Figure S34. Seasonal mean bias in daily average $PM_{2.5}$ for 2019 at AQS monitoring sites for CRACMM1. The annotations in the lower left show the mean (min, max) site average biases.

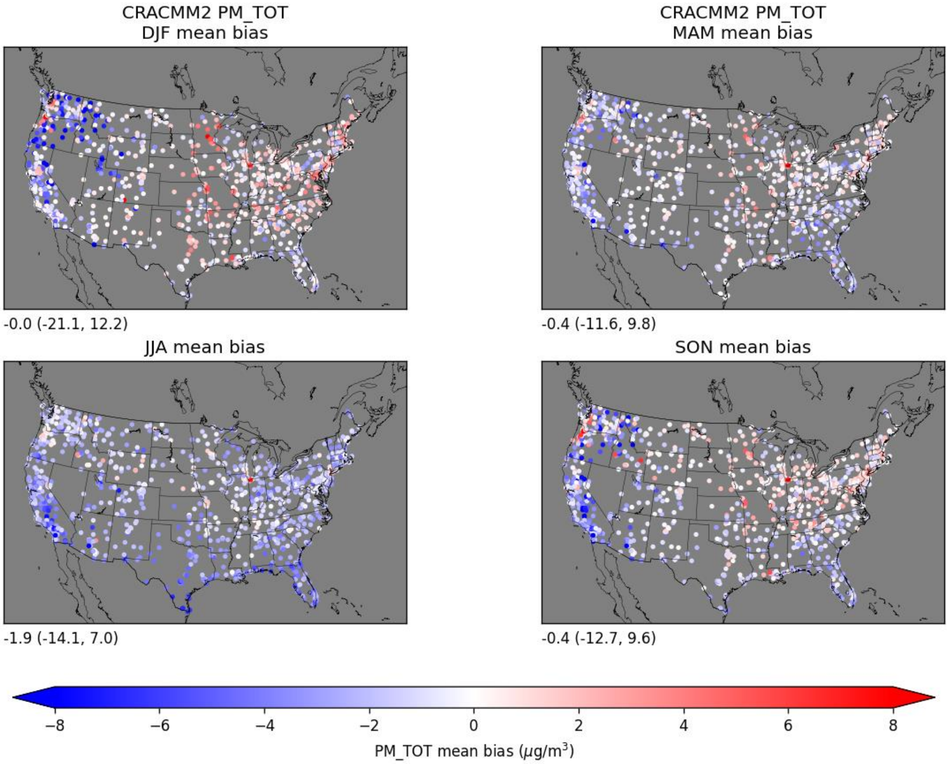

Figure S35. Seasonal mean bias in daily average $PM_{2.5}$ for 2019 at AQS monitoring sites for CRACMM2. The annotations in the lower left show the mean (min, max) site average biases.

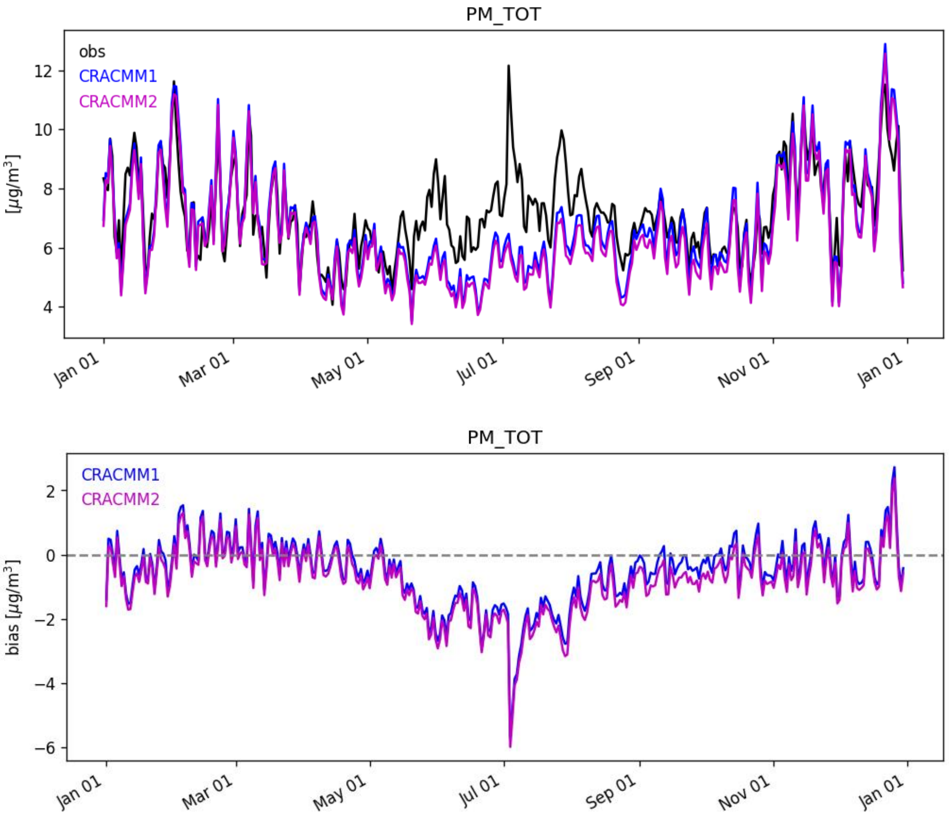

Figure S36. Daily average $PM_{2.5}$ (top) and bias (bottom) for 2019 averaged over all AQS monitoring sites for CRACMM1 and CRACMM2.

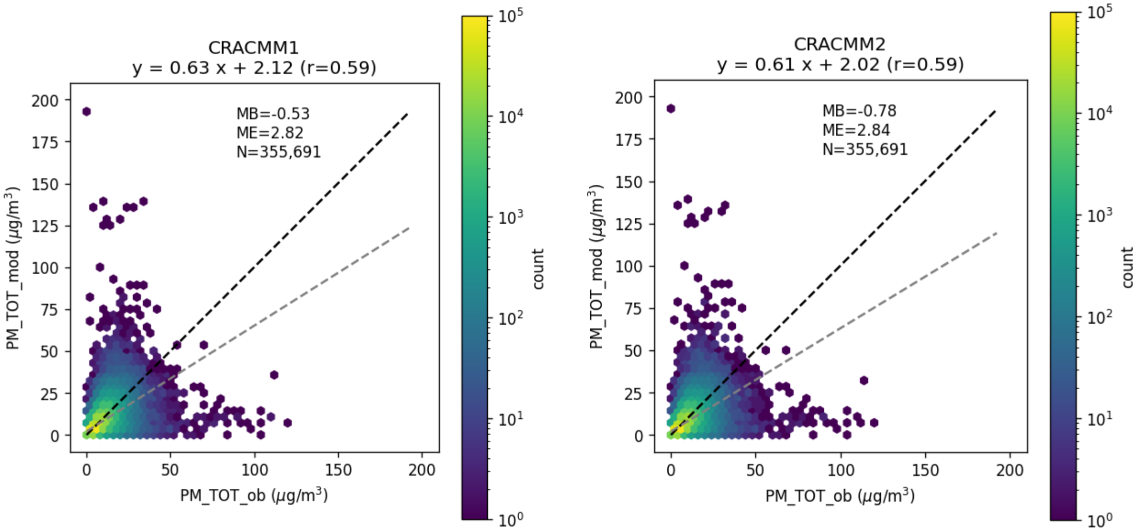

Figure S37. Density scatter plot of observed daily average $PM_{2.5}$ vs. CRACMM1 (left) and CRACMM2 (right). The mean bias (MB), mean error (ME), and number of observations (N) are indicated on each panel.

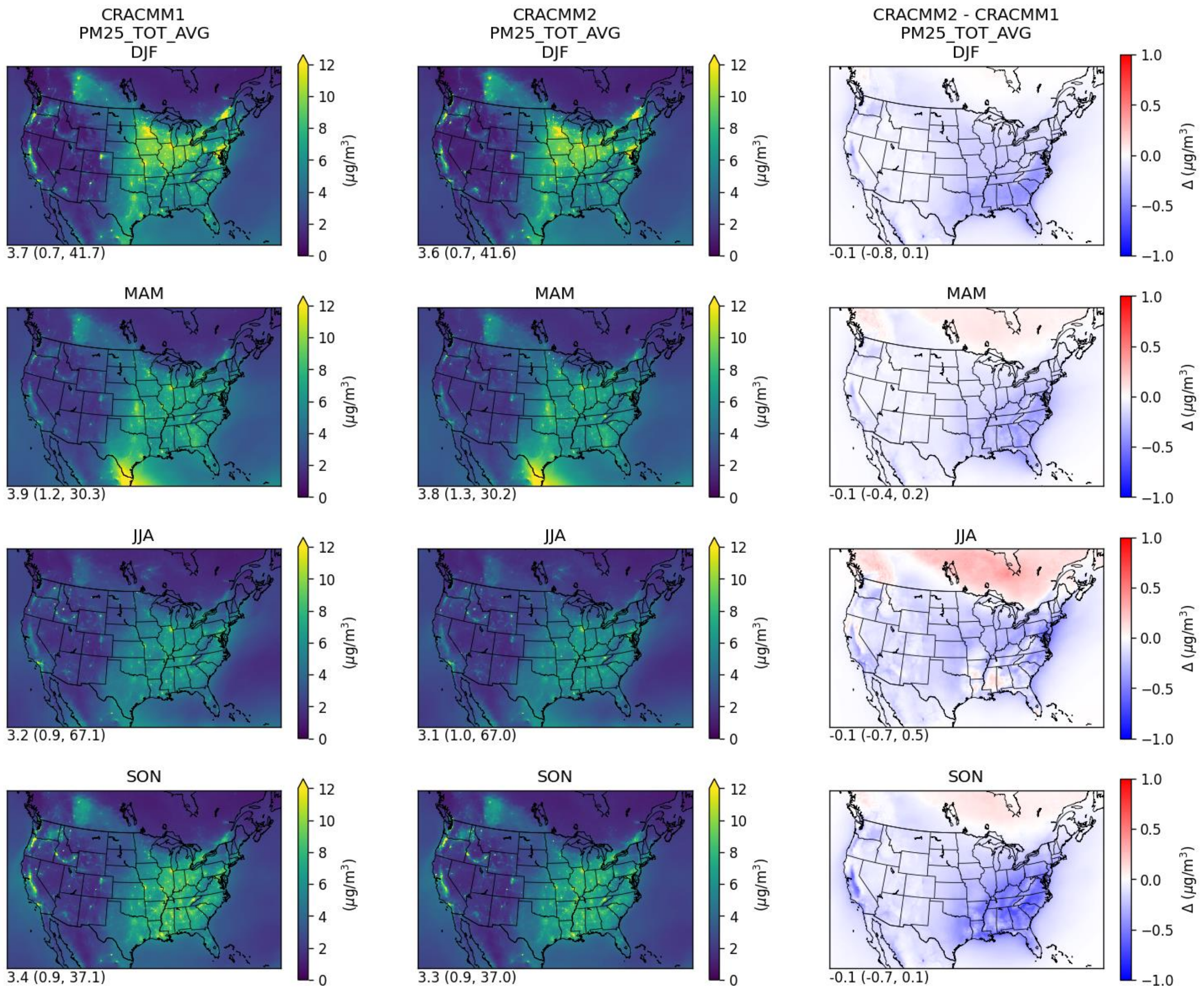

Figure S38. Seasonal average $PM_{2.5}$ in CRACMM1 (left column) and CRACMM2 (middle column) and the change in CRACMM2 compared to CRACMM1 (right column). The annotations in the lower left show the mean (min, max) over the modeling domain.

435

References

Browne, E. C., Wooldridge, P. J., Min, K. E., and Cohen, R. C.: On the role of monoterpene chemistry in the remote continental boundary layer, Atmos. Chem. Phys., 14, 1225-1238, 10.5194/acp-14-1225-2014, 2014.

Fisher, J. A., Jacob, D. J., Travis, K. R., Kim, P. S., Marais, E. A., Chan Miller, C., Yu, K., Zhu, L., Yantosca, R. M., Sulprizio, M. P., Mao, J., Wennberg, P. O., Crounse, J. D., Teng, A. P., Nguyen, T. B., St. Clair, J. M., Cohen, R. C., Romer, P., Nault, B. A., Wooldridge, P. J., Jimenez, J. L., Campuzano-Jost, P., Day, D. A., Hu, W., Shepson, P. B., Xiong, F., Blake, D. R., Goldstein, A. H., Misztal, P. K., Hanisco, T. F., Wolfe, G. M., Ryerson, T. B., Wisthaler, A., and Mikoviny, T.: Organic nitrate chemistry and its implications for nitrogen budgets in an isoprene- and monoterpene-rich atmosphere: constraints from aircraft (SEAC4RS) and ground-based (SOAS) observations in the Southeast US, Atmos. Chem. Phys., 16, 5969-5991, 10.5194/acp-16-5969-2016, 2016.

Ivatt, P. D., Evans, M. J., and Lewis, A. C.: Suppression of surface ozone by an aerosol-inhibited photochemical ozone regime, Nature Geoscience, 15, 536-540, 10.1038/s41561-022-00972-9, 2022.

Jacob, D. J.: Heterogeneous chemistry and tropospheric ozone, Atmospheric Environment, 34, 2131-2159, https://doi.org/10.1016/S1352-2310(99)00462-8, 2000.

Jaffe, D. A., Ninneman, M., Nguyen, L., Lee, H., Hu, L., Ketcherside, D., Jin, L., Cope, E., Lyman, S., Jones, C., O'Neil, T., and Mansfield, M. L.: Key results from the salt lake regional smoke, ozone, and aerosol study (SAMOZA), Journal of the Air & Waste Management Association, 74, 163-180, 10.1080/10962247.2024.2301956, 2024.

KNMI: S5P/TROPOMI HCHO ATBD, 2022.

Mouat, A. P., Siegel, Z. A., and Kaiser, J.: Evaluation of Aeris mid-infrared absorption (MIRA), Picarro CRDS (cavity ring-down spectroscopy) G2307, and dinitrophenylhydrazine (DNPH)-based sampling for long-term formaldehyde monitoring efforts, Atmos. Meas. Tech., 17, 1979-1994, 10.5194/amt-17-1979-2024, 2024.

Pye, H. O. T., Luecken, D. J., Xu, L., Boyd, C. M., Ng, N. L., Baker, K. R., Ayres, B. R., Bash, J. O., Baumann, K., Carter, W. P. L., Edgerton, E., Fry, J. L., Hutzell, W. T., Schwede, D. B., and Shepson, P. B.: Modeling the Current and Future Roles of Particulate Organic Nitrates in the Southeastern United States, Environmental Science & Technology, 49, 14195-14203, 10.1021/acs.est.5b03738, 2015.

Schwantes, R. H., Emmons, L. K., Orlando, J. J., Barth, M. C., Tyndall, G. S., Hall, S. R., Ullmann, K., St. Clair, J. M., Blake, D. R., Wisthaler, A., and Bui, T. P. V.: Comprehensive isoprene and terpene gas-phase chemistry improves simulated surface ozone in the southeastern US, Atmos. Chem. Phys., 20, 3739-3776, 10.5194/acp-20-3739-2020, 2020.

U.S. EPA: Technical Support Document (TSD): Preparation of Emissions Inventories for the 2019 North American Emissions Modeling Platform, 2022.

Vereecken, L. and Nozière, B.: H migration in peroxy radicals under atmospheric conditions, Atmos. Chem. Phys., 20, 7429-7458, 10.5194/acp-20-7429-2020, 2020.

Wennberg, P. O., Bates, K. H., Crounse, J. D., Dodson, L. G., McVay, R. C., Mertens, L. A., Nguyen, T. B., Praske, E., Schwantes, R. H., Smarte, M. D., St Clair, J. M., Teng, A. P., Zhang, X., and Seinfeld, J. H.: Gas-Phase Reactions of Isoprene and Its Major Oxidation Products, Chemical Reviews, 118, 3337-3390, 10.1021/acs.chemrev.7b00439, 2018.

Wiser, F., Place, B. K., Sen, S., Pye, H. O. T., Yang, B., Westervelt, D. M., Henze, D. K., Fiore, A. M., and McNeill, V. F.: AMORE-Isoprene v1.0: a new reduced mechanism for gas-phase isoprene oxidation, Geosci. Model Dev., 16, 1801-1821, 10.5194/gmd-16-1801-2023, 2023.

Yang, B., Wiser, F. C., McNeill, V. F., Fiore, A. M., Tao, M., Henze, D. K., Sen, S., and Westervelt, D. M.: Implementation and evaluation of the automated model reduction (AMORE) version 1.1 isoprene oxidation mechanism in GEOS-Chem, Environmental Science: Atmospheres, 3, 1820-1833, 10.1039/D3EA00121K, 2023.

Zhu, Q., Schwantes, R. H., Coggon, M., Harkins, C., Schnell, J., He, J., Pye, H. O. T., Li, M., Baker, B., Moon, Z., Ahmadov, R., Pfannerstill, E. Y., Place, B., Wooldridge, P., Schulze, B. C., Arata, C., Bucholtz, A., Seinfeld, J. H., Warneke, C., Stockwell, C. E., Xu, L., Zuraski, K., Robinson, M. A., Neuman, J. A., Veres, P. R., Peischl, J., Brown, S. S., Goldstein, A. H., Cohen, R. C., and McDonald, B. C.: A better representation of volatile organic compound chemistry in WRF-Chem and its impact on ozone over Los Angeles, Atmos. Chem. Phys., 24, 5265-5286, 10.5194/acp-24-5265-2024, 2024.
